# Performance of Polypropylene Fiber-Reinforced Mortar Exposed to Elevated Temperatures

**DOI:** 10.12688/f1000research.175541.1

**Published:** 2026-01-08

**Authors:** Murtatha Alshijlawi, Sheelan Mahmoud Hama, Musab A. Abdulhamed, Ibraheem A Aidan, Noor A Rajab, Aymen Hameed Fayyadh

**Affiliations:** 1 Anbar Technical Institute, Middle Technical University, Baghdad, Baghdad Governorate, Iraq; 2 Department of Civil Engineering, University of Anbar, Ramadi, Al Anbar Governorate, Iraq; 3 Department of Civil Engineering, University of Al Maarif, Ramadi, Anbar, Iraq

**Keywords:** load-deflection, toughness, residual strength, high temperatures, polypropylene fibers, and mortar

## Abstract

**Background:**

Cement-based materials deteriorate significantly when exposed to high temperatures due to extensive microcracking, increased porosity, and dehydration of hydration products, all of which lower the materials’ mechanical performance and post-fire stability. This makes thermally robust mortar essential for protective applications in fire-prone areas as well as structural restoration.

**Method:**

The goal of this study is to assess how the amount of polypropylene (PP) fiber in cement mortar affects its mechanical behavior, thermal resistance, and residual performance at temperatures as high as 600°C. Prior to and following exposure to increased temperatures of 200, 400, and 600°C, mortar mixes containing 0%, 0.5%, 1.0%, and 1.5% PP fibers by volume were made and tested for workability, density, compressive strength, flexural strength, and flexural toughness.

**Results:**

The results show that increasing PP fiber content decreases workability. PP fibers significantly improved high-temperature performance. At 600°C, the control mix retained only 19% of its compressive strength, while the 0.5% PP mix retained 33%. Flexural strength increased by 26–44% at ambient temperature for 0.5–1.0% PP fiber content, and at 600°C, PP mixes preserved up to 8% more flexural strength than the control. Toughness improved substantially, with the 1.5% PP mix showing nearly 10-time higher residual toughness than the control at 600°C.

**Conclusions:**

In conclusion, the incorporation of 0.5-1.0% polypropylene fibers provides a balanced enhancement in thermal resistance, residual strength and ductility, confirming its effectiveness in producing mortar with superior structural integrity after exposed to high temperatures.

## 1. Introduction

One of the most important cementitious materials in building, mortar is used extensively in masonry, plastering, restoration projects, and ferrocement slabs. ^1, 2^ Its resilience to high temperatures is still a major worry, though, especially for buildings that have been subjected to fire. High temperatures cause a number of physical and chemical changes in mortar that impair its ability to provide structural integrity. Physically, mass loss, increased porosity, and widespread microcracking result from the dehydration of calcium silicate hydrate (C–S–H) and calcium hydroxide at higher temperatures, followed by the evaporation of free water below 100°C. ^3– 5^ Around 400–500°C, calcium hydroxide breaks down chemically into calcium oxide and water, but the C–S–H gel’s gradual decalcification permanently changes the microstructure. ^6, 7^ Compressive, flexural, and tensile strengths are significantly reduced as a result of these changes, and in extreme situations, explosive spalling that reveals reinforcement and hastens structure collapse occurs. ^8, 9^ Husem ^8^ found sharp reduction in compressive and flexural strength at 600°C. One of solution to improve the behavior of concrete at ambient and high-temperatures is adding fibers to concrete or mortar. ^10, 11^ Kalifa et al. ^10^ and Zeiml et al. ^12^ showed that PP fibers reduce spalling due to vapor-release channels formed during melting. Because PP fibers have a relatively low melting temperature (160–170°C), they melt and soften when heated, forming tiny channels in the mortar. By creating pathways for the release of vapor, these channels lower internal pore pressure and lessen the possibility of explosive spalling. ^10, 12^ PP fibers increase fracture energy, flexural toughness, and crack resistance at room temperature and at slightly higher temperatures, which increases durability. ^13, 14^ Alvarez et al. ^15^ and Shahriar et al. ^16^ showed that high PP contents increase porosity after melting and may reduce residual strength. PP fibers help keep strength at lower content and moderate temperatures (up to 400 ^o^ C), but the voids that generated when the fibers melt and lose their ability to bridge may cause residual strength to decrease after cooling. ^15– 17^ Although, concrete has been studied extensively; mortar shows different pore structure, cracking modes, and thermal sensitivity, as emphasized by Mindeguia et al. ^18^ and Yermak et al., ^19^ most previous studies focus on PP fiber-reinforced concrete, but this work systematically investigates mortar, which has different pore structure, thermal sensitivity, and cracking mechanisms. To address these gaps, this study evaluates the high-temperature performance of mortar reinforced with 0–1.5% PP fibers, assessing density, compressive strength, flexural strength, load–deflection behavior, and toughness up to 600°C. The findings provide mortar-specific insights into fiber–matrix interactions under thermal exposure and identify the fiber dosage that delivers optimal post-fire mechanical integrity.

This study provides mortar-specific experimental evidence and mechanistic understanding of how PP fibers influence strength retention, crack control, toughness, and thermal resistance up to 600°C, identifying the optimal fiber dosage for fire-exposed mortar and offering quantitative data not previously available in the literature.

## 2. Experimental work

### 2.1 Materials, mix proportions, mixing and casting procedure

Ordinary Portland Cement, conforming to Iraqi specification (I.S.) No. 5 ^20^ have been used. The used natural river sand has maximum particle size of 4.25 mm, specific gravity of 2.60, finesse of 2.50, SO _3_% of 0.15, and absorption% of 0.68%. The size distribution and properties of used sand complied with I.S. No. 45. ^21^ Potable water free from impurities, used for all mixes and curing. PP fibers with a density of approximately 0.91 kg/l, and melting point between 160–170°C, length 12 mm, and diameter 18 μm, respectively have been used.

Mixtures were prepared with a constant water/cement ratio of 0.45 and cement: sand ratio of 1:3 by weight. Polypropylene fibers were incorporated at dosages of 0.0%, 0.5%, 1.0%, and 1.5% by volume, the maximum percentages 1.5% used because more than this value the fibers will cause blocking and agglomeration. A 1% “superplasticizer type F” has been added to keep the mixes workable. The mixes proportions have been listed in Table 1.

**Table 1.** Mixes proportional.

PP fiber content (% by volume)	Cement (kg/m ^3^)	Sand (kg/m ^3^)	Water (kg/m ^3^)	Superplasticizer (% of cement)
0.0%	550	1650	247.5	1%
0.5%	550	1650	247.5	1%
1.0%	550	1650	247.5	1%
1.5%	550	1650	247.5	1%

Dry materials (cement, sand, fibers) were mixed before adding water gradually. Fresh mortar was cast into molds, compacted in two layers, and surface finished. Specimens were demolded after 24 hours and cured in water at 20 ± 2°C until the age of 28 days. Prior to thermal exposure, all specimens were oven-dried at 105°C for 24 hours to remove free water and minimize explosive spalling risk.

### 2.2 Testing and temperature exposure

**Figure 1.**
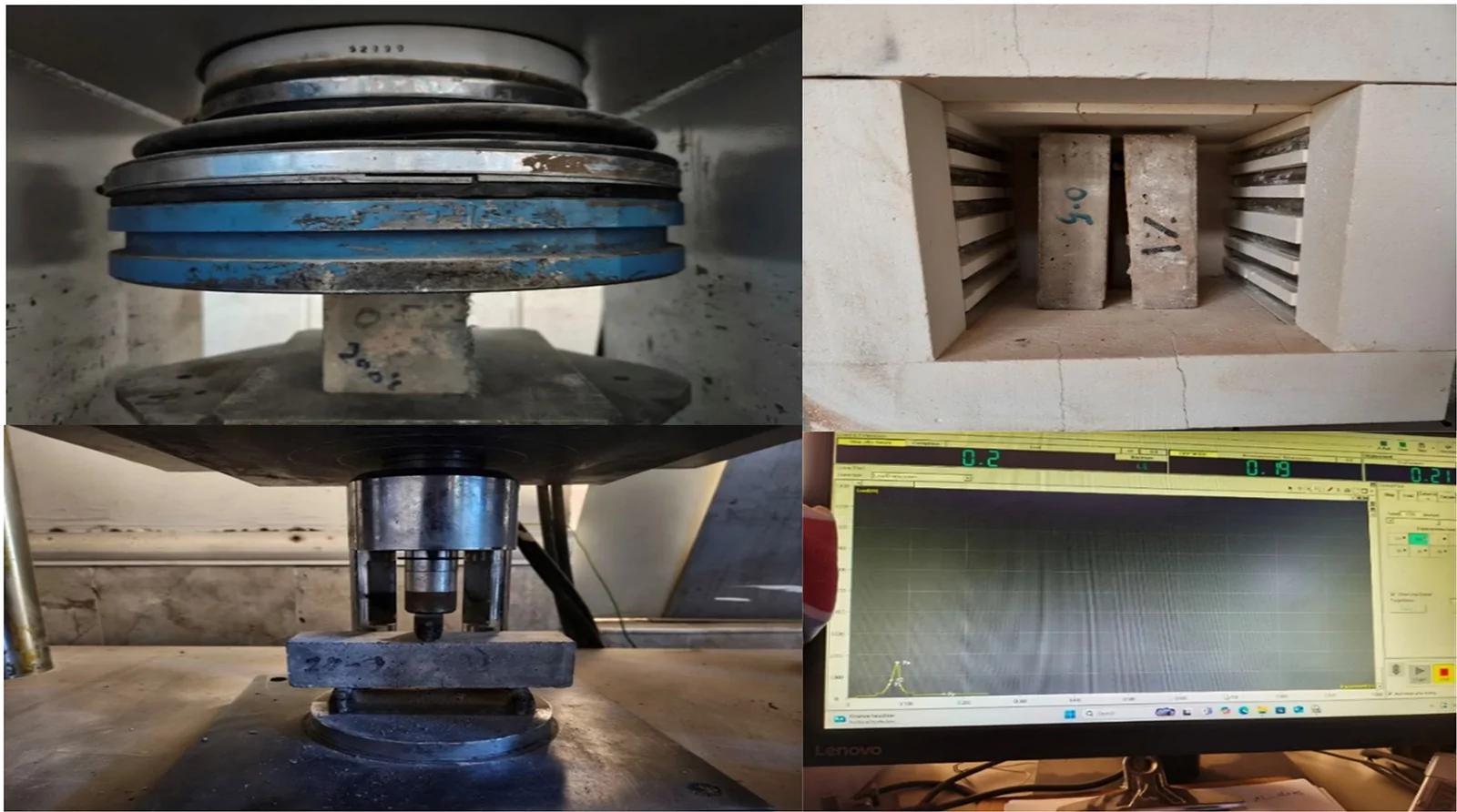
Tests conducted in this study.

**Figure 2.**
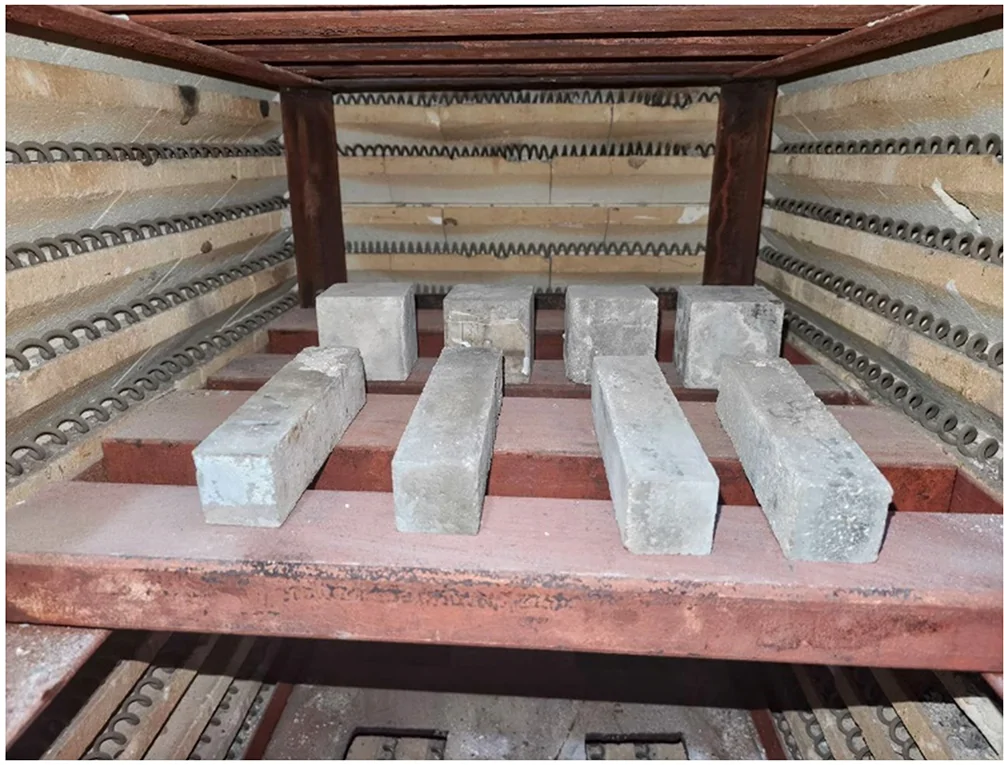
The process of applying high temperatures to specimens.

**Figure 3.**
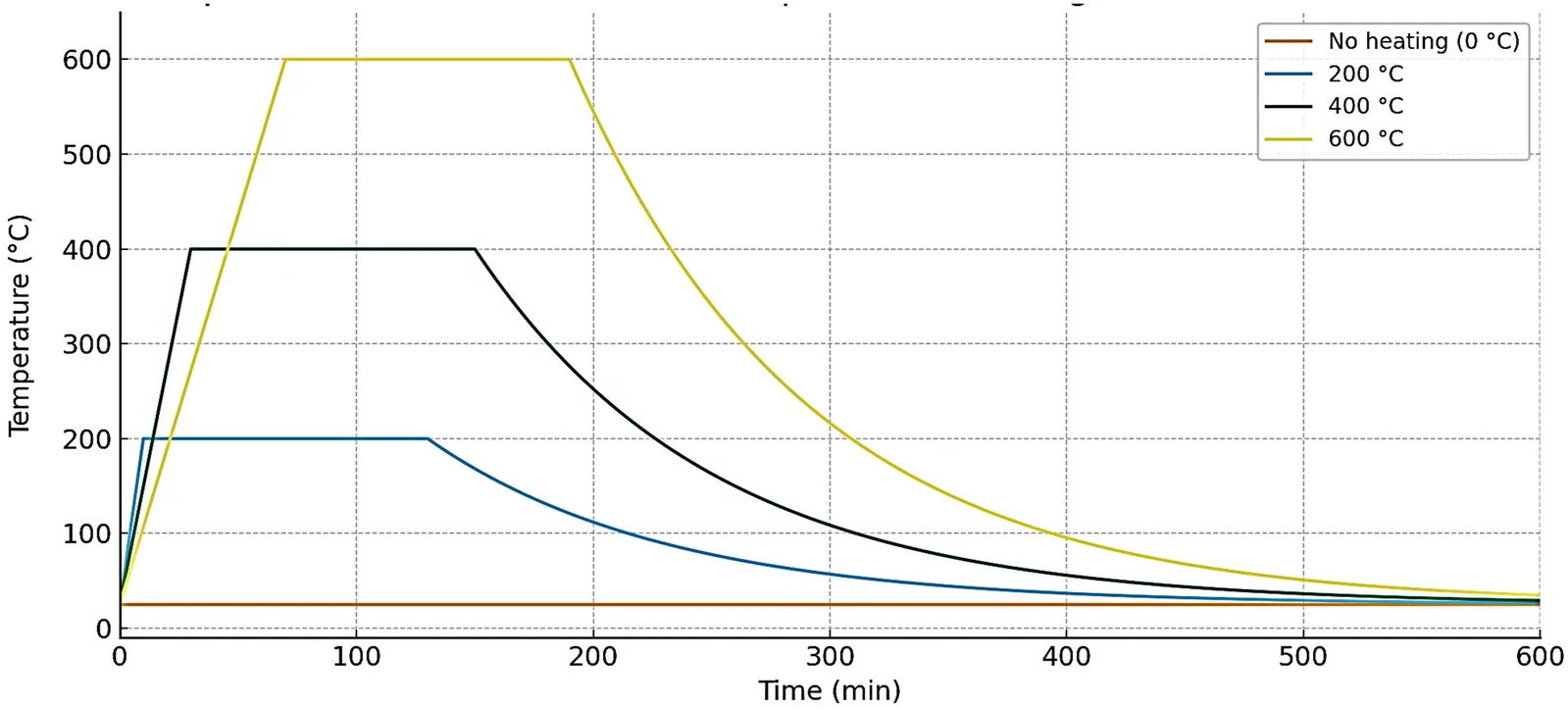
The temperatures profiles.

Flow Table Test was made according to ASTM C1437. ^22^ The lubricated flow mold was placed at the center of the table and filled with fresh mortar in two layers. The mold was lifted vertically after 15 seconds. The table was dropped 25 times in 15 seconds. The final spread diameter was measured along two perpendicular axes and averaged. Compressive and flexural strength were measured according to ASTM C109 ^23^ and ASTM C348, ^24^ respectively. Compressive strength was measured on three 50 × 50 × 50 mm cubes for each mix, using a 3000 kN compression machine. Load was applied continuously at 2400 ± 200 N/s until failure. The maximum load was recorded, and compressive strength was calculated. Three cubes were tested for each mix and averaged value have been adopted. Flexural strength was evaluated using three 50 × 50 × 160 mm prisms for each mix, under three-point loading. The prism was positioned with 100 mm clear span. Load was applied at 50 ± 10 N/s through third-point loading. Peak load at failure was recorded, and flexural strength was calculated. Load–deflection behavior was measured during flexural testing to evaluate post-cracking response and toughness. Dry density residual % was calculated as the percentage reduction in specimen mass after thermal exposure, the average of results of three specimens has been adopted, Figure 1 shows the tests that have been made in this study. Concrete specimens were exposed to elevated temperatures of 200, 400, and 600°C in an electric furnace, see Figure 2, in accordance with ISO 834 ^25^ and EN 1363-1 ^26^ fire testing requirements. Heating was applied at a controlled rate of approximately 5°C/min until the designated target temperature was reached. Figure 3 shows the temperature–time profile followed during the heating and cooling stages of the thermal exposure process. This figure is essential because it shows that the heating rate, target temperatures, and cooling regime strictly followed the ISO 834 standard fire curve, as required in international fire-resistance testing. ^25, 26^ Each target temperature was maintained under isothermal conditions for a duration of 2 h to ensure thermal equilibrium throughout the specimens. Following the heating stage, the furnace was switched off and the specimens were subjected to natural cooling inside the furnace chamber under ambient laboratory conditions, simulating the post-fire cooling regime recommended in the standard. The cooling process followed the characteristic nonlinear decay prescribed by ISO 834, whereby the temperature decreases progressively toward ambient at a diminishing rate, thereby reproducing realistic post-fire exposure conditions.

## 3. Results and Discussion

### 3.1 Slump flow

**Figure 4.**
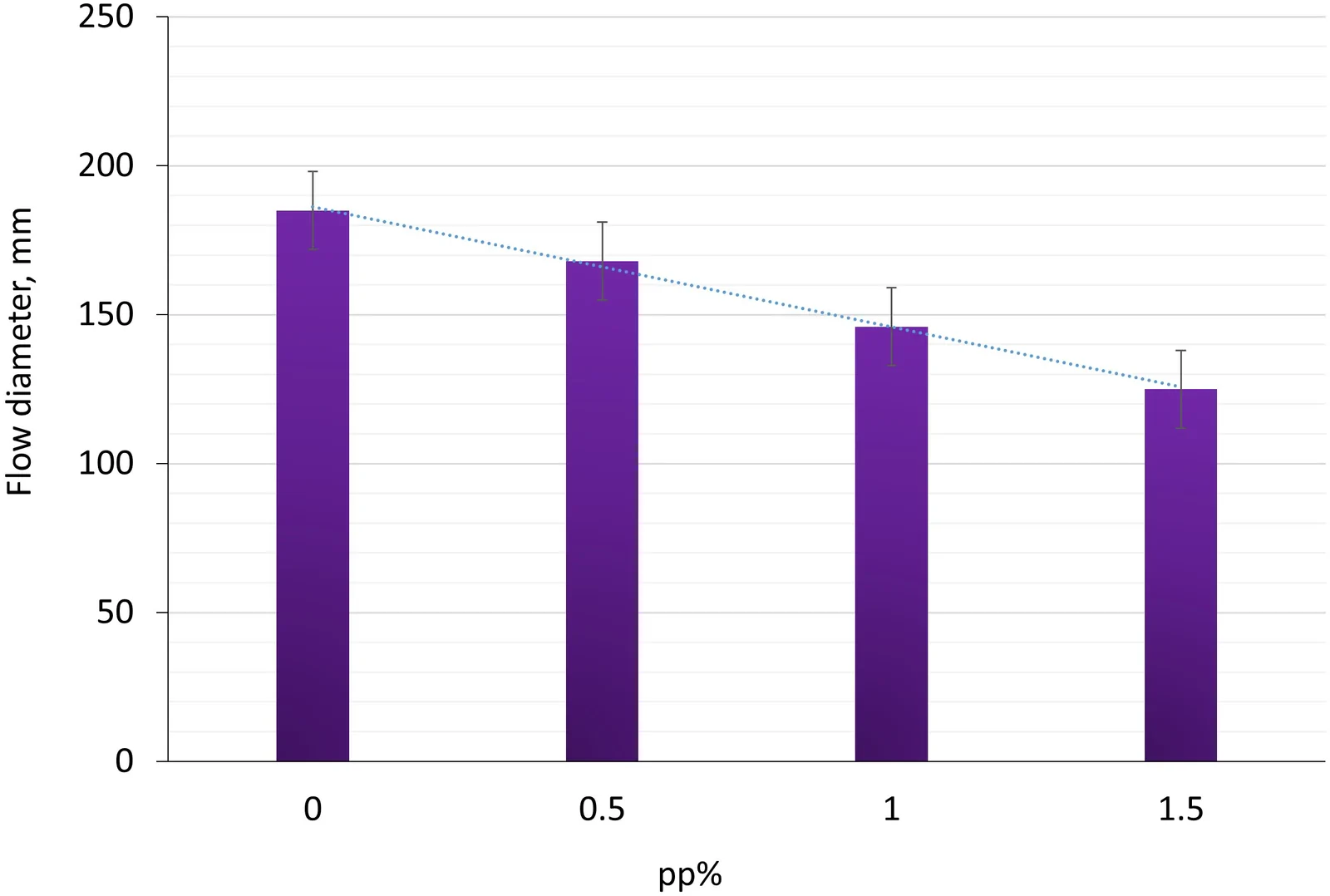
The flow diameter vs. pp%.

The data clearly shows a decrease in the workability of the mortar as the Polypropylene (PP) fiber content increases, see Figure 4. The flow diameter a measure of workability drops from 185 mm for the control mix to 125 mm 1.5% PP fibers.

The flow diameter reduction in this study about 32.4%, which is nearly identical to the 25–35% reduction documented by Jawad and Al-Haydari ^27^ and Kumar et al. ^28^ confirming that workability reduction is a predictable consequence of increased PP fiber content.

The addition of a large number of fine PP fibers significantly increases the total surface area within the mix. Since the water/cement ratio is typically kept constant, the available free water (the water lubricating the particles) is reduced, leading to a drier, stiffer mix and lower workability. Also, the dispersed fibers create an internal network or “skeletal structure” within the mortar. This network physically interlocks the cement and sand particles, hindering their relative movement and sliding.

### 3.2 Density

**Figure 5.**
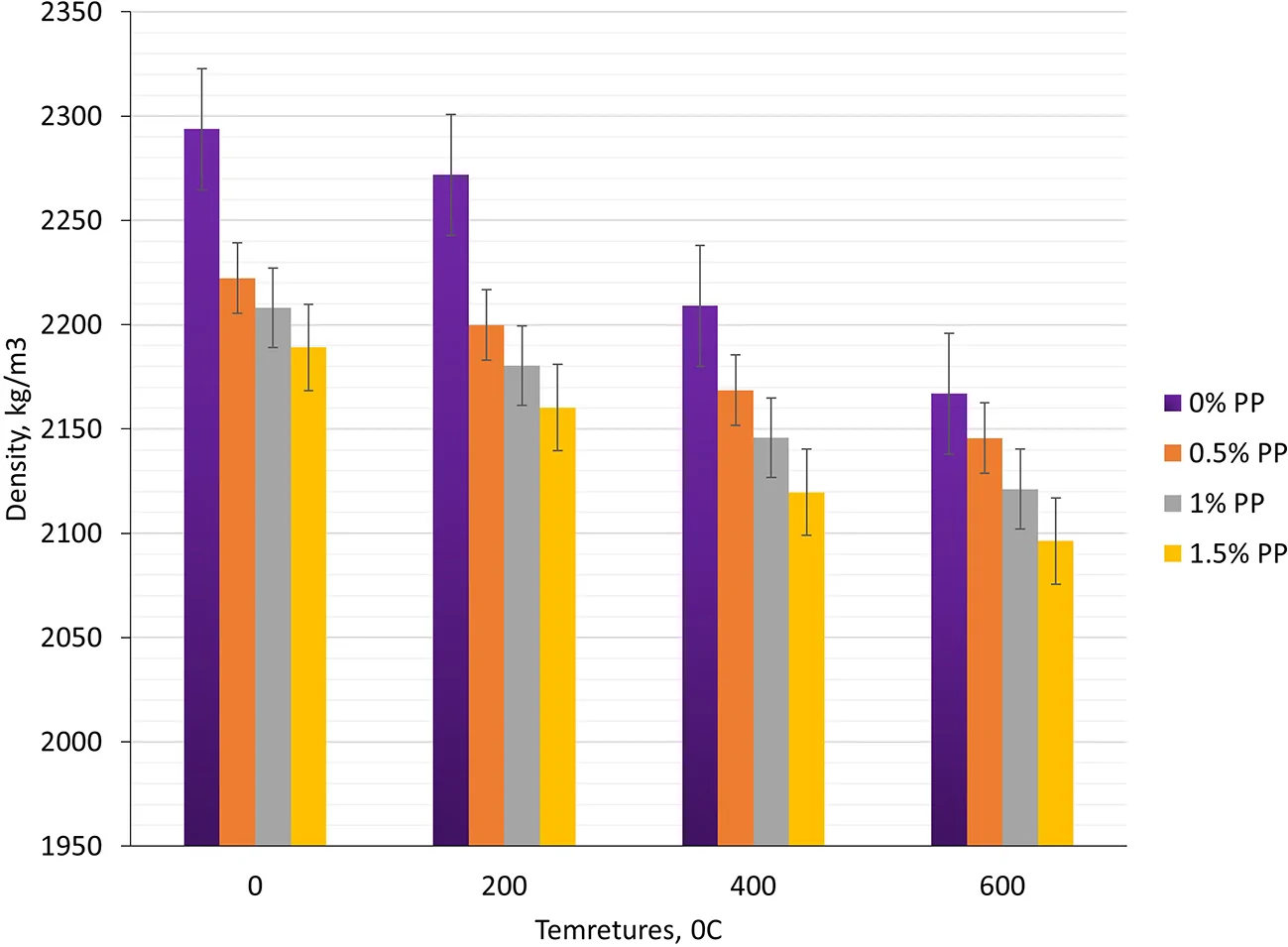
The density vs. pp% for different temperatures level.

Results are shown in Figure 5 and Table 2. As illustrated in Figure 5, the density of all mortar mixtures decreased progressively with the increase in temperature. This decline can be attributed to the evaporation of physically and chemically bound water, the decomposition of hydration products, and the development of internal microcracks within the matrix. At room temperature (25°C), the density values ranged between 2189 and 2293 kg/m ^3^ depending on the fiber content.

**Table 2.** Dry density and residual density percentage at various pp% and temperature levels.

PP fiber content (%)	Temperature (°C)	Dry Density (kg/m ^3^)	Residual %
0.0	0	2293.74	100.0
	200	2271.87	99.0
	400	2209.05	96.3
	600	2166.91	94.5
0.5	0	2222.33	100.0
	200	2199.84	99.0
	400	2168.55	97.6
	600	2145.62	96.5
1.0	0	2208.12	100.0
	200	2180.41	98.7
	400	2145.77	97.2
	600	2121.18	96.0
1.5	0	2189.08	100.0
	200	2160.30	98.7
	400	2119.63	96.8
	600	2096.32	95.8

In comparison to the reference condition, the densities decreased by roughly 5–6% when the temperature rose to 600°C. Xiao and König ^29^ reported similar low density loss, suggesting that strength degradation is dominated by microstructural degeneration rather than bulk mass loss. They found 5–7% density loss for concrete at 600°C. Because PP fibers have a lower specific gravity than cementitious components, their incorporation somewhat decreased the initial density. At higher temperatures, though, the variations between the combinations lost some of their significance. This suggests that while the fibers might have assisted in maintaining structural integrity by slowing the spread of cracks, they had little effect on the mortar matrix’s overall thermal degradation trend.

It can be observed from Table 2 that all mixes maintained over 94% of their original density up to 600°C. The control mix shows a slightly sharper reduction, reaching 94.5% retention, whereas the mixes with PP fibers residual strength was between 95.8% and 96.5% compared to control one. This improvement in density retention with the inclusion of PP fibers can be related to the fibermelting mechanism, which forms micro-channels allowing vapor escape and minimizing internal pressure buildup. Consequently, this mechanism mitigates explosive spalling and microcrack propagation, resulting in slightly better dimensional and mass stability under thermal exposure. Overall, the results suggest that adding PP fibers enhances the mortar’s ability to maintain its compactness and resist degradation at elevated temperatures.

### 3.3 Compressive strength

**Figure 6.**
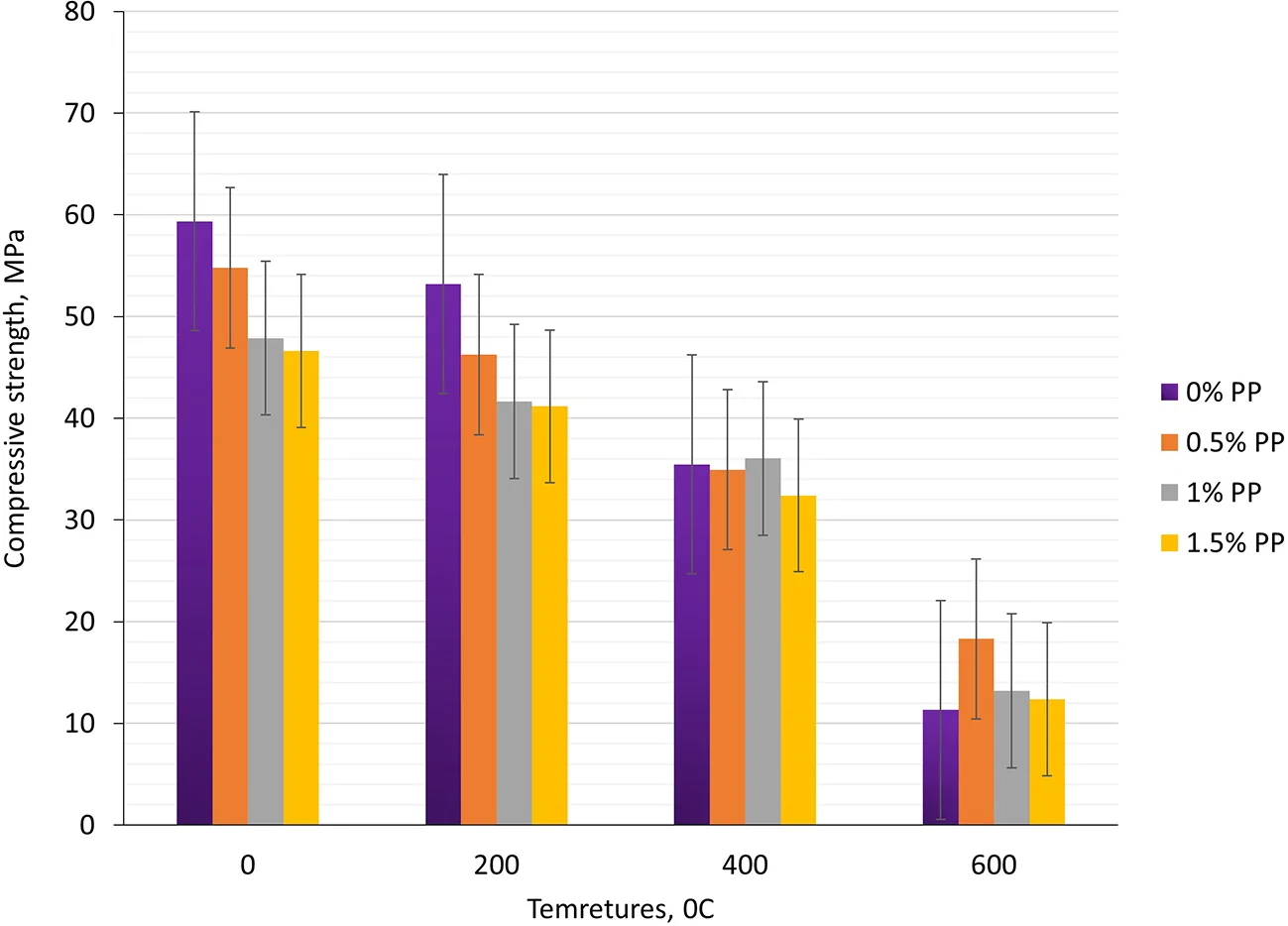
Compressive strength vs. temperature for different PP fiber contents.

**Figure 7.**
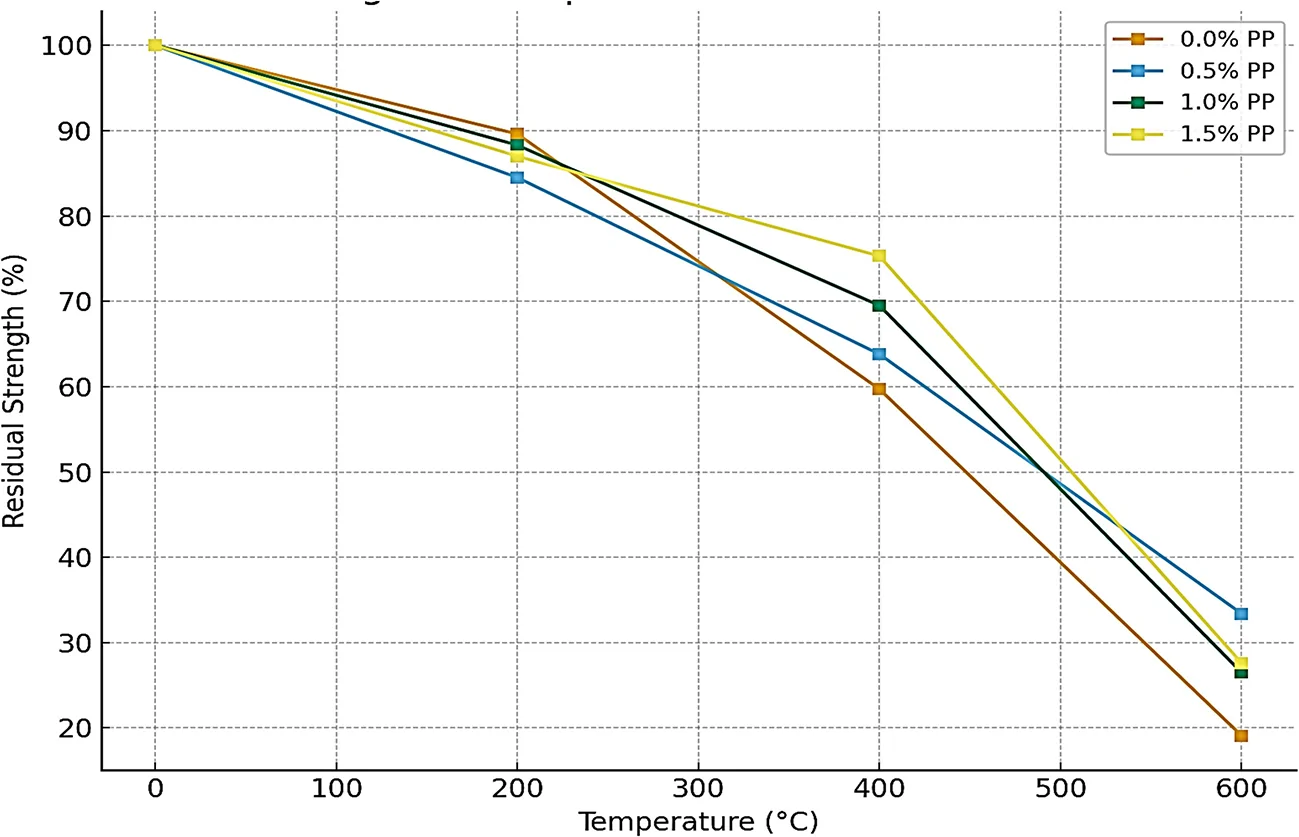
Residual strength vs. temperature for various PP fiber contents.

The Figures 6, 7 and Table 3 reports compressive strength for mixtures with 0, 0.5, 1.0 and 1.5% PP fibers after exposure to 0, 200, 400 and 600°C with Residual Strength (%).

**Table 3.** The compressive and residual strength% for different pp% at different temperatures.

PP fiber content (%)	Temperature (°C)	Compressive strength (MPa)	Residual strength (%)
0.0	0	59.36	100.0
	200	53.19	89.6
	400	35.46	59.7
	600	11.32	19.1
0.5	0	54.77	100.0
	200	46.26	84.5
	400	34.95	63.8
	600	18.30	**33.4**
1.0	0	47.88	100.0
	200	41.65	87.0
	400	36.04	75.3
	600	13.21	27.6
1.5	0	46.61	100.0
	200	41.16	88.3
	400	32.41	69.5
	600	12.36	26.5

As shown in Figure 6, the compressive strength of all mortar specimens decreased progressively with increasing temperature, demonstrating the thermal degradation of the cementitious matrix. PP fibers slightly reduce compressive strength at ambient temperature due to increased porosity, their value lies in high-temperature performance, not initial strength. Upon heating, the fibers melt to produce pressure-relief channels that reduce spalling and limit microcracking. As a result, fiber-reinforced mortars retain much higher residual strength and toughness than the control mix, demonstrating that the benefit of fibers is post-fire structural integrity, not compressive strength at room temperature. ^30^ At 200°C, the reduction in strength was moderate (approximately 10–15%), primarily due to the evaporation of free water and the initiation of microcracking caused by thermal expansion mismatch between the cement paste and aggregates. ^30, 31^ Beyond 400°C, a sharp decline in strength was observed, particularly for the control mixture, which residual only about 60% of its initial strength. This behavior corresponds to the decomposition of calcium silicate hydrate (C–S–H) gel and partial dehydration of calcium hydroxide (CH), both of which weaken the matrix cohesion. ^32, 33^

The control mix showed significant deterioration at 600°C, keeping only 19% of its original strength. On the other hand, mortars reinforced with PP fiber, particularly those that contain 0.5% and 1.5% PP, maintain a comparatively higher strength (33–28%, respectively). The melting of PP fibers at 160–170°C creates micro-channels that release internal vapor pressure, preventing explosive spalling and internal damage and contributing to the enhanced performance of fiber-reinforced specimens. ^34, 35^ This process slows the propagation of thermal cracks and improves the matrix’s structural integrity.

Husem ^6^ found residual strength of 20–22% for mortar at 600°C, while Alvarez et al. ^15^ found 30–35% residual strength for PP-reinforced mortar. Shahriar et al. ^16^ found 32–36% residual strength at 600°C for PP-fiber concrete.

The residual compressive strength% vs. exposure to high temperatures is shown in Figure 7. The results show that at all temperature levels over 200°C, specimens with PP fibers demonstrated higher residual strength when compared to the control mix. In this case, the 1.5% PP mixture’s residual strength at 400°C was almost 75%, while the control’s was 59.7%. Likewise, the 0.5% PP mixture strength remained 33.4% of the control mix strength at 600°C.

This increased residual strength is in line with results from earlier research that showed PP fibers enhance cementitious composites’ high temperature resistance and post-heating recovery through encouraging vapor release, lowering pore pressure, and lessening the likelihood of explosive spalling. ^36, 37^ Additionally, the distributed fibers maintain improved load transfer inside the composite by acting as crack arresters throughout both heating and cooling cycles. ^38^ Overall, the findings show that a moderate amount of PP fibers (about 0.5% to 1.0%) can effectively reduce heat damage and improve mortar’s mechanical recovery after fire. But because to poor dispersion or increased porosity, an excessively high fiber content may somewhat lower the initial compressive strength.

### 3.4 Load–displacement curves

**Figure 8.**
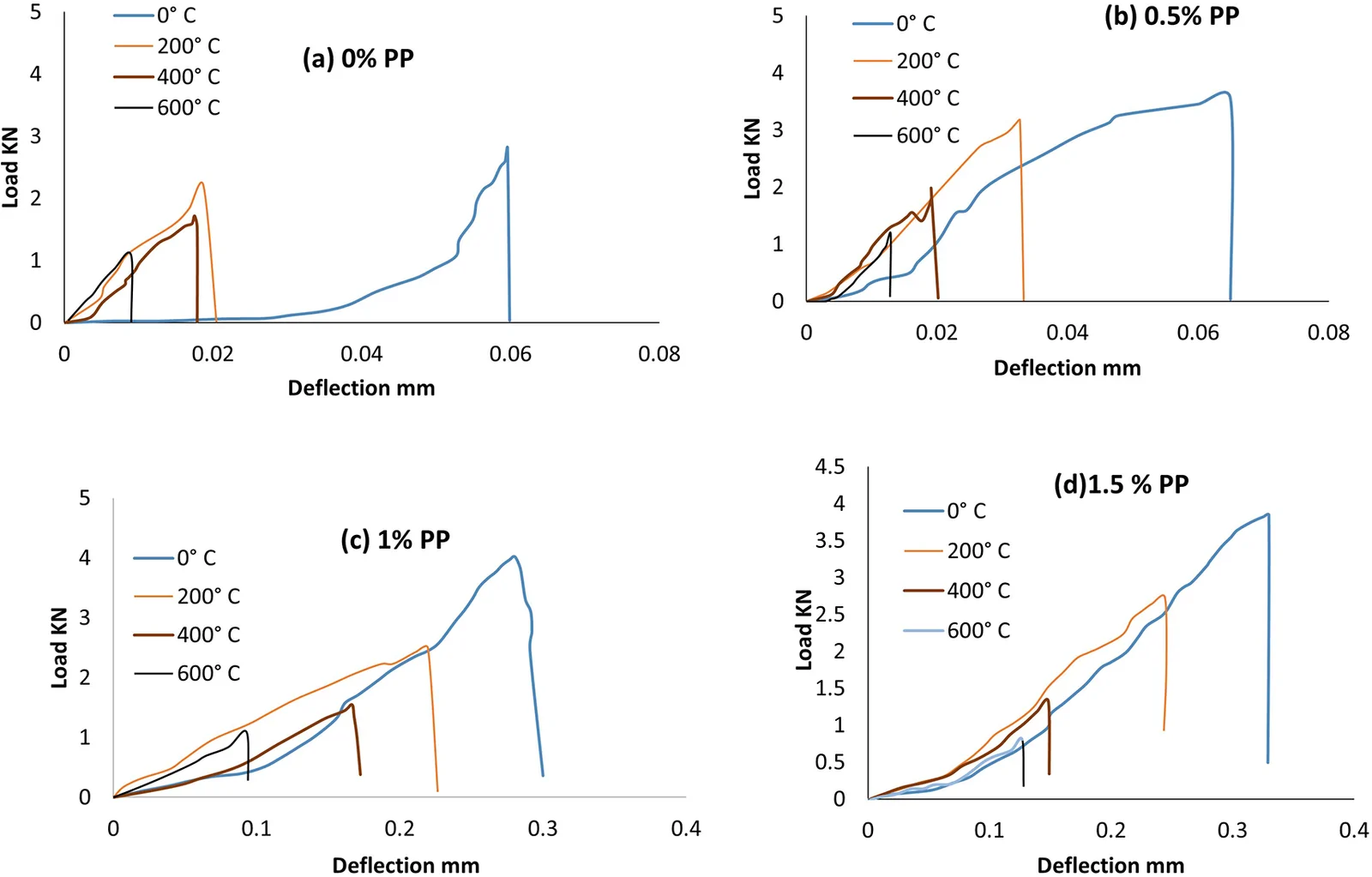
Load – Deflection vs. temperature for different PP fiber contents.

Both the ultimate load-carrying capacity (Pmax) and the deflection at failure (Δ) are clearly impacted by the PP fiber content and exposure temperature, according to the findings of the flexural tests shown in Figure 8 and Table 4. Flexural strength and ductility were significantly reduced (Δ dropped from 0.0600 mm to 0.0068 mm) in the fiber-free reference mix, which showed a steady decrease in Pmax from 2.80 kN at room temperature to 1.09 kN at 600°C. At all temperature settings, the addition of PP fibers improved the deformation capacity and load resistance. The blend with 0.5% PP fibers demonstrated better thermal stability by achieving a greater initial load capacity of 3.55 kN and maintaining 3.17 kN at 200°C.

**Table 4.** The ultimate load and maximum recorded deflection at failure for different pp% for different temperatures level.

PP fiber content (%)	Temperature (°C)	Pmax (kN)	Δ at failure (mm)
0.0%	0	2.800	0.0600
	200	2.200	0.0205
	400	1.529	0.0180
	600	1.090	0.0068
0.5%	0	3.550	0.0650
	200	3.173	0.0330
	400	1.970	0.0600
	600	1.180	0.0130
1.0%	0	4.020	0.2800
	200	2.492	0.3000
	400	1.550	0.1660
	600	1.0952	0.0920
1.5%	0	3.820	0.3290
	200	2.730	0.2440
	400	1.325	0.1480
	600	0.857	0.1170

Despite fiber softening, this mix maintained a significant amount of ductility at 400°C (Δ = 0.0600 mm), suggesting efficient crack-bridging and stress redistribution. The deflection at ambient temperature (0.3 mm) and Δ at 200°C (0.2200 mm) for the fiber content to 1.0% resulted in a significant improvement in deformability compared to control mia, which mean improved post-cracking energy absorption due to fiber plasticization.

For fiber content (1.5% PP) a decreased in thermal resistance and load capacity have been notice, which may be result of pore development and fiber aggregation after melting. Due to the degradation of the cement matrix and the loss of fiber–matrix interaction, all mixtures exhibited a decrease in strength and ductility at high temperatures (400–600°C).

PP fibers are known to soften at temperatures between 160–170°C and melt near 320–340°C, generating a micro-network of channels that relieve vapor pressure within the cementitious matrix and reduce internal cracking and explosive spalling. ^7, 38– 40^ At moderate temperatures (≤400°C), these fibers undergo partial softening, which promotes crack bridging and energy dissipation, thereby enhancing deflection capacity, as observed in the 0.5% and 1.0% PP mixes.

Higher temperatures (>400°C) cause the fibers to completely melt, which weakens the matrix–fiber interface and creates pores, lowering stiffness and load capacity. ^41^ The dehydration of C-S-H gel, the decarbonation of calcium carbonate, and the formation of microcracks are all responsible for the decrease in residual load capacity at 600°C that was seen across all fiber compositions. ^6– 8^ Despite this deterioration, PP fibers, especially when used at the optimal dosage of 0.5%, significantly postpone the loss of stiffness and maintain residual ductility by improving stress redistribution following cracking. A balanced fiber content can give synergistic increases in both thermal resistance and post-failure deformation capacity, according to similar findings reported by other researchers studying PP fiber-reinforced concretes exposed to heat. ^9– 11^

### 3.5 Flexural strength

**Figure 9.**
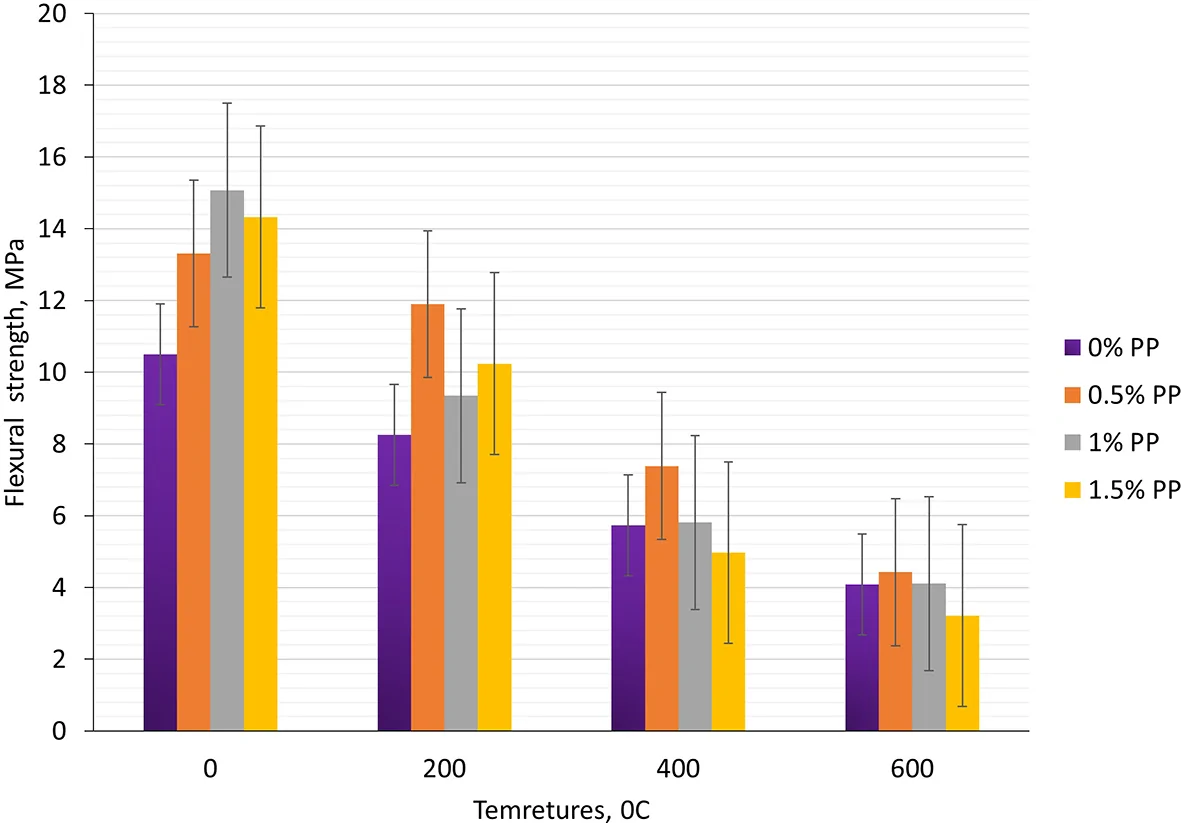
Flexural strength vs. temperature for different PP fiber contents.

The relationship between temperature and flexural strength (fr) for mortars with varying PP fiber concentrations is depicted in Figure 9. The σ of the control mix gradually decreased, going from 10.5 MPa at 25°C to 4.1 MPa at 600°C, or a 61% decrease. By achieving 13.3 MPa at 0.5% PP and 14.7 MPa at 1.0% PP, the addition of PP fibers greatly increased strength under ambient conditions, indicating a higher stress transfer through fiber bridging and decreased microcrack propagation. ^8, 33, 37^ All mixtures, however, demonstrated significant strength loss at 200°C and beyond as a result of matrix micro cracking, cement hydrate dehydration, and polymer melting. ^7, 32, 35^

The 0.5% PP mix retains about 42% of its original strength at 400°C, but the 1.0% and 1.5% PP mixes retain 38% and 35% of their initial strength, respectively. Fiber melting and pore formulation caused further deterioration above 400°C. ^3, 42^ However, fiber-reinforced mixtures continued to perform better than unreinforced mortar, demonstrating the usefulness of PP in redistributing stress prior to melting. The percentage change in σ with temperature is shown in Table 5, which shows that the highest relative loss happens above 400°C. According to the trend, polymer combustion takes precedence over the crack-bridging action above a threshold temperature of around 350 to 400°C. ^43^ The area under the load-displacement curve, or estimated toughness, shows that the use of PP significantly improves post-peak energy absorption.

**Table 5.** Flexural strength vs. PP% at different temperatures.

PP fiber content (%)	Temperature (°C)	f _r_ (MPa)	% change
0.0%	0	10.5000	—
0.5%	0	13.3125	+26.83%
1.0%	0	15.0750	+43.57%
1.5%	0	14.3250	+36.43%
0.0%	200	8.2500	—
0.5%	200	11.8988	+44.25%
1.0%	200	9.3450	+13.27%
1.5%	200	10.2375	+24.09%
0.0%	400	5.7338	—
0.5%	400	7.3875	+28.85%
1.0%	400	5.8125	+1.37%
1.5%	400	4.9688	−13.34%
0.0%	600	4.0875	—
0.5%	600	4.4250	+8.26%
1.0%	600	4.1070	+0.48%
1.5%	600	3.2138	−21.38%

The flexural strength retention observed in the present study (55% for 0.5% PP and 38–42% for other mixes at 400°C) aligns well with the 30–45% range reported by Noumowé ^42^ and the 35–50% range noted by Li et al. ^43^ These quantitative similarities confirm that PP-reinforced mortar exhibits a similar degradation trend to PP-reinforced concrete under thermal loading.

### 3.6 Toughness

**Figure 10.**
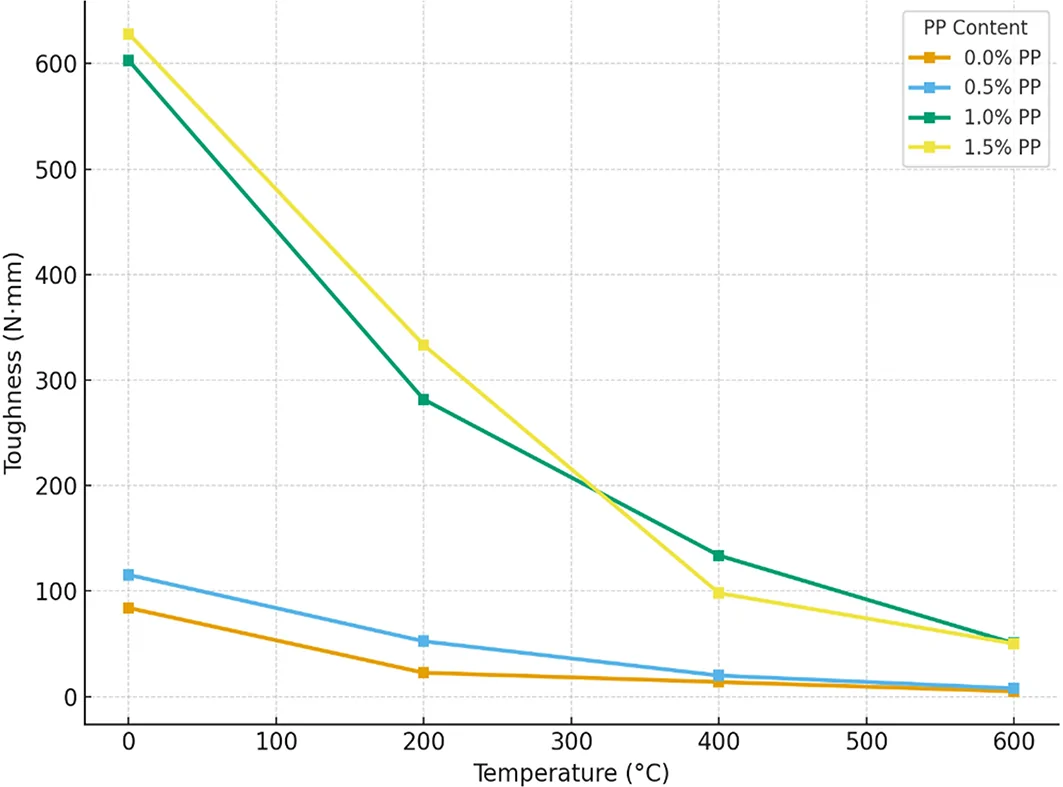
Flexural toughness vs. temperature for different PP fiber contents.

The flexural toughness has been calculated from area under load-displacement curves and the results illustrated in Figure 10. For these results, at ambient temperature, the flexural toughness rose from 0.052 N·mm/mm ^2^ for control specimen to 0.072 N·mm/mm ^2^ for specimens contain 0.5% PP, resulted in higher ductility and better deformation capacity. This improvement is in line with recent studies that demonstrate that lower dosages of PP encourage matrix–fiber synergy, but higher dosages may result in fiber aggregation and voids. ^44, 45^ Up to 1.0% PP addition often improves flexural performance and heat resistance at mild temperatures (≤400°C); however, structural pore expansion and polymer disintegration cause significant degradation above this range. ^37, 45^ At elevated temperatures, at 600°C, the 1.5% PP mix retained a residual toughness of 50 N·mm, which was 10 times higher than the control, indicating maintained post-cracking deformation capability. ^43^

These results are in line with prior studies showing that post-peak energy dissipation in high-temperature concrete is greatly enhanced by small PP additions (0.5–1.0%). ^46^ While maintaining exceptional thermal stability, too much PP (>1.0%) may cause voids that compromise matrix integrity at room temperature. ^9, 43^ Overall, the toughness trends demonstrate that PP fibers are helpful in lowering heat-induced brittleness and enhancing residual performance by improving ductility and energy absorption under both normal and thermal stress settings.

## 4. Conclusion

This study examined the mechanical performance and thermal resistance of mortar containing 0–1.5% PP fibers exposed to temperatures up to 600°C. The following conclusions have been drawn based on results:

1. Incorporating PP fibers decreased flow diameter from 185 mm (0% PP) to 125 mm (1.5% PP), representing a 32.4% reduction, primarily due to increased internal friction and surface area.
2. All mixtures retained above 94% of their original density at 600°C. The control mix kept 94.5%, whereas PP fiber mixes retained slightly higher values (96.0–96.5%), confirming that melted fibers help limit microcrack propagation.
3. At ambient temperature, compressive strength decreased with fiber inclusion (from 59.36 MPa for the control to 46.61–54.77 MPa for PP mixes.
4. At 600°C, however, PP fibers improved residual strength, for control mix was 19.1% residual strength while for 0.5% PP was 33.4% residual strength (75% improvement over control), 26.5% for 1.0% PP, and 27.6% for 1.5% PP.
5. At ambient temperature, flexural strength increased significantly with PP fibers.
6. After exposure to 600°C, fiber-reinforced mixes kept 0.5–8.3% more flexural strength compared to the control.
7. Flexural toughness improved markedly due to fiber bridging. At 600°C, the 1.5% PP mix retained toughness values nearly 10 times higher than the control, confirming substantial enhancement in post-cracking energy absorption.
8. Based on results of all tested made in this study, 0.5% PP fibers provided the best balance between initial performance and high-temperature residual behavior, offering the highest combined improvement in residual compressive and flexural, and ductility.

## Data Availability

The datasets supporting the finding of this study are openly available in Zenodo: Data Manuscript: Performance of Polypropylene Fiber-Reinforced Mortar Exposed to Elevated Temperatures repository at https://doi.org/10.5281/zenodo.18056598. ^47^ This project contains the following data:
•
The data avialability file.xlsb Data are available under the terms of the Creative Commons Attribution 4.0 International license (CC-BY 4.0).
