# Performance of Polypropylene Fiber-Reinforced Mortar Exposed to Elevated Temperatures

**DOI:** 10.12688/f1000research.175541.2

**Published:** 2026-03-19

**Authors:** Murtatha Alshijlawi, Sheelan Mahmoud Hama, Musab A. Abdulhamed, Ibraheem A Aidan, Noor A Rajab, Aymen Hameed Fayyadh

**Affiliations:** 1 Anbar Technical Institute, Middle Technical University, Baghdad, Baghdad Governorate, Iraq; 2 Department of Civil Engineering, University of Anbar, Ramadi, Al Anbar Governorate, Iraq; 3 Department of Civil Engineering, University of Al Maarif, Ramadi, Anbar, Iraq

**Keywords:** load-deflection, toughness, residual strength, high temperatures, polypropylene fibers, and mortar

## Abstract

**Background:**

Cement-based materials deteriorate significantly when exposed to high temperatures due to extensive microcracking, increased porosity, and dehydration of hydration products, all of which lower the materials’ mechanical performance and post-fire stability. This makes thermally robust mortar essential for protective applications in fire-prone areas as well as structural restoration.

**Method:**

The goal of this study is to assess how the amount of polypropylene (PP) fiber in cement mortar affects its mechanical behavior, thermal resistance, and residual performance at temperatures as high as 600°C. Prior to and following exposure to increased temperatures of 200, 400, and 600°C, mortar mixes containing 0%, 0.5%, 1.0%, and 1.5% PP fibers by volume were made and tested for workability, density, compressive strength, flexural strength, and flexural toughness.

**Results:**

The results show that increasing PP fiber content decreases workability. PP fibers significantly improved high-temperature performance. At 600°C, the control mix retained only 19% of its compressive strength, while the 0.5% PP mix retained 33%. Flexural strength increased by 26–44% at ambient temperature for 0.5–1.0% PP fiber content, and at 600°C, PP mixes preserved up to 8% more flexural strength than the control. Toughness improved substantially, with the 1.5% PP mix showing nearly 10-time higher residual toughness than the control at 600°C.

**Conclusions:**

In conclusion, the incorporation of 0.5-1.0% polypropylene fibers provides a balanced enhancement in thermal resistance, residual strength and ductility, confirming its effectiveness in producing mortar with superior structural integrity after exposed to high temperatures.

## 1. Introduction

One of the most important cementitious materials in building, mortar is used extensively in masonry, plastering, restoration projects, and ferrocement slabs. ^1, 2^ Its resilience to high temperatures is still a major worry, though, especially for buildings that have been subjected to fire. High temperatures cause a number of physical and chemical changes in mortar that impair its ability to provide structural integrity. Physically, mass loss, increased porosity, and widespread microcracking result from the dehydration of calcium silicate hydrate (C–S–H) and calcium hydroxide at higher temperatures, followed by the evaporation of free water below 100°C. ^3– 5^ Around 400–500°C, calcium hydroxide breaks down chemically into calcium oxide and water, but the C–S–H gel’s gradual decalcification permanently changes the microstructure. ^6, 7^ Compressive, flexural, and tensile strengths are significantly reduced as a result of these changes, and in extreme situations, explosive spalling that reveals reinforcement and hastens structure collapse occurs. ^8, 9^ Husem ^8^ found sharp reduction in compressive and flexural strength at 600°C. One of solution to improve the behavior of concrete at ambient and high-temperatures is adding fibers to concrete or mortar. ^10, 11^ Kalifa et al. ^10^ and Zeiml et al. ^12^ showed that PP fibers reduce spalling due to vapor-release channels formed during melting. Because PP fibers have a relatively low melting temperature (160–170°C), they melt and soften when heated, forming tiny channels in the mortar. By creating pathways for the release of vapor, these channels lower internal pore pressure and lessen the possibility of explosive spalling. ^10, 12^ PP fibers increase fracture energy, flexural toughness, and crack resistance at room temperature and moderate temperatures, thereby improving the durability and deformation capacity of cementitious composites. ^13, 14^ Alvarez et al. ^15^ and Shahriar et al. ^16^ showed that high PP contents increase porosity after melting and may reduce residual strength. PP fibers help keep strength at lower content and moderate temperatures (up to 400°C), but the voids that generated when the fibers melt and lose their ability to bridge may cause residual strength to decrease after cooling. ^15– 17^ Although, concrete has been studied extensively; mortar shows different pore structure, cracking modes, and thermal sensitivity, as emphasized by Mindeguia et al. ^18^ and Yermak et al. ^19^ Most previous studies focus on PP fiber-reinforced concrete, but this work systematically investigates mortar, which has different behavior mechanisms. To address these gaps, this study evaluates the high-temperature performance of mortar reinforced with 0–1.5% PP fibers, assessing density, compressive strength, flexural strength, load–deflection behavior, and toughness up to 600°C. Unlike most previous studies that focus on concrete, this work provides one of the few systematic investigations of polypropylene fiber-reinforced cement mortar subjected to temperatures up to 600°C with combined evaluation of strength, ductility, and toughness.

## 2. Experimental work

### 2.1 Materials, mix proportions, mixing and casting procedure

Ordinary Portland Cement, conforming to Iraqi specification (I.S.) No. 5 ^20^ have been used. The used natural river sand has maximum particle size of 4.25 mm, specific gravity of 2.60, finesse of 2.50, SO _3_% of 0.15, and absorption% of 0.68%. The size distribution and properties of used sand complied with I.S. No. 45. ^21^ Potable water free from impurities, used for all mixes and curing. PP fibers with a density of approximately 0.91 kg/l, and melting point between 160–170°C, length 12 mm, and diameter 18 μm, respectively have been used.

A polycarboxylate-based high-range water-reducing admixture (HRWR), Sika ViscoCrete-180 GS, conforming to ASTM C494 Type F, was used to improve the workability of the mortar mixtures. The superplasticizer has a specific gravity of approximately 1.07, pH value ranging from 4 to 6, and a light brownish liquid appearance. The admixture is based on modified polycarboxylate ether technology and was added at a constant dosage of 1% by weight of cement for all mixtures to maintain comparable workability conditions while isolating the effect of polypropylene fiber content on the fresh and hardened properties of the mortar.

Mixtures were prepared with a constant water/cement ratio of 0.45 and cement: sand ratio of 1:3 by weight. Polypropylene fibers were incorporated at dosages of 0.0%, 0.5%, 1.0%, and 1.5% by volume, the maximum percentages 1.5% used because more than this value the fibers will cause blocking and agglomeration. The mixes proportions have been listed in Table 1.

**Table 1.** Mixes proportional.

PP fiber content (% by volume)	Cement (kg/m ^3^)	Sand (kg/m ^3^)	Water (kg/m ^3^)	Superplasticizer (% of cement)
0.0%	550	1650	247.5	1%
0.5%	550	1650	247.5	1%
1.0%	550	1650	247.5	1%
1.5%	550	1650	247.5	1%

Dry materials (cement, sand, fibers) were mixed before adding water gradually. Fresh mortar was cast into molds, compacted in two layers, and surface finished. After demolding at 24 h, the specimens were immersed in tap water maintained at 20 ± 2°C until the age of 28 days. The curing water was periodically renewed to maintain stable curing conditions throughout the curing period. Prior to thermal exposure, all specimens were oven-dried at 105°C for 24 hours to remove free water and minimize explosive spalling risk.

### 2.2 Testing and temperature exposure

Flow Table Test was made according to ASTM C1437. ^22^ Compressive and flexural strength were measured according to ASTM C109 ^23^ and ASTM C348, ^24^ respectively. Compressive strength was measured on three 50 × 50 × 50 mm cubes for each mix, using a 3000 kN compression machine. Load was applied continuously at 2400 ± 200 N/s until failure. The maximum load was recorded, and compressive strength was calculated. The flexural tests were performed using a three-point bending configuration on prism specimens (50 × 50 × 160 mm) in accordance with ASTM C348. The tests were conducted using a universal testing machine with a capacity of 300 kN, and the loading was applied under load control at a constant rate of 50 ± 10 N/s, as specified in the standard. During testing, the load–deflection response was recorded to evaluate post-cracking behavior and flexural toughness. Figure 1 shows the tests that have been made in this study. Concrete specimens were exposed to elevated temperatures of 200, 400, and 600°C in an electric furnace, see Figure 2, in accordance with ISO 834 ^25^ and EN 1363-1 ^26^ fire testing requirements. Heating was applied at a controlled rate of approximately 5°C/min until the designated target temperature was reached. Figure 3 shows the temperature–time profile followed during the heating and cooling stages of the thermal exposure process. This figure is essential because it shows that the heating rate, target temperatures, and cooling regime strictly followed the ISO 834 standard fire curve, as required in international fire-resistance testing. ^25, 26^ Each target temperature was maintained under isothermal conditions for a duration of 2 h to ensure thermal equilibrium throughout the specimens. Following the heating stage, the furnace was switched off and the specimens were subjected to natural cooling inside the furnace chamber under ambient laboratory conditions, simulating the post-fire cooling regime recommended in the standard. The cooling process followed the characteristic nonlinear decay prescribed by ISO 834, whereby the temperature decreases progressively toward ambient at a diminishing rate, thereby reproducing realistic post-fire exposure conditions. Summary of specimen details and testing standards used in the experimental program has been illustrated in Table 2.

**Figure 1.**
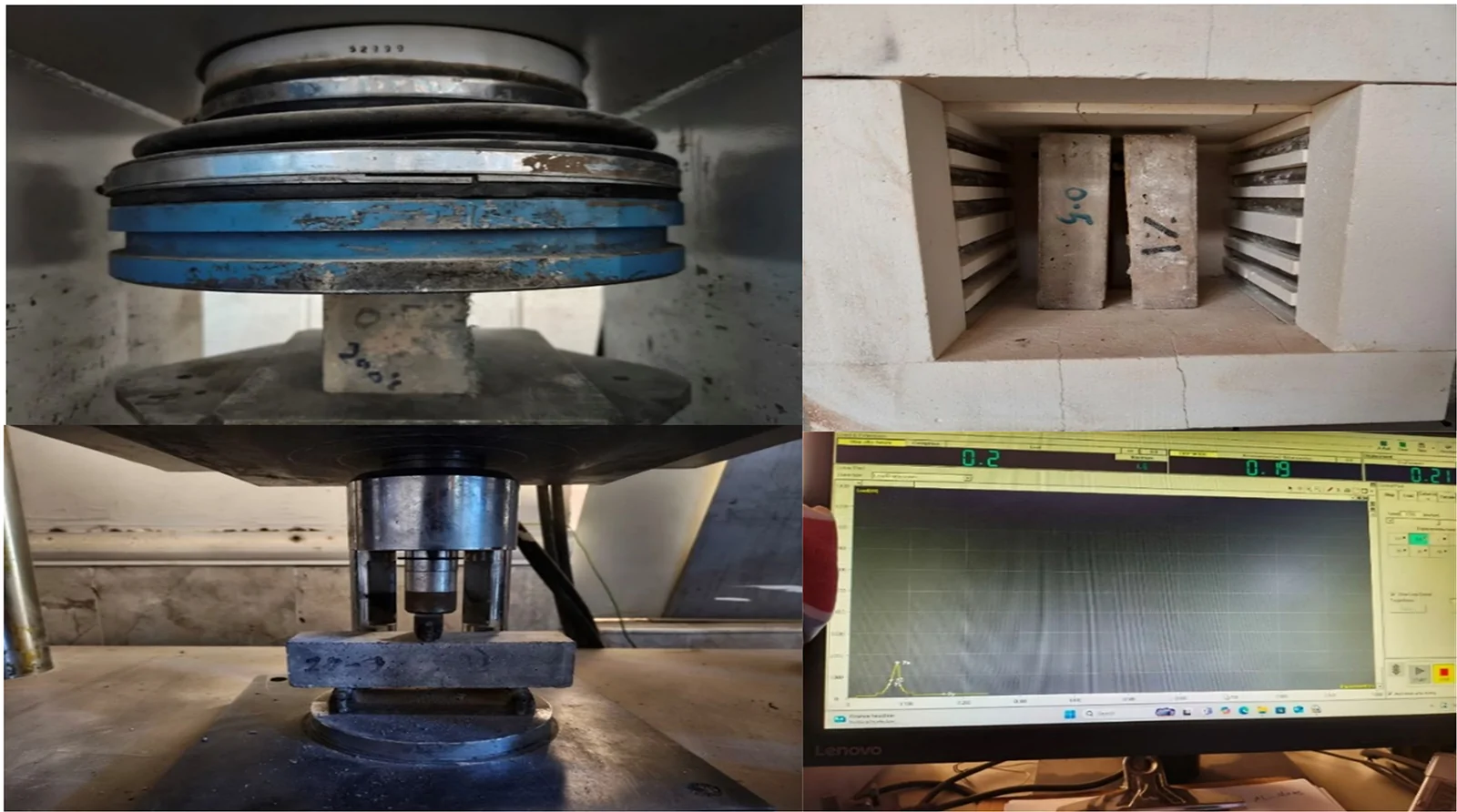
Tests conducted in this study.

**Figure 2.**
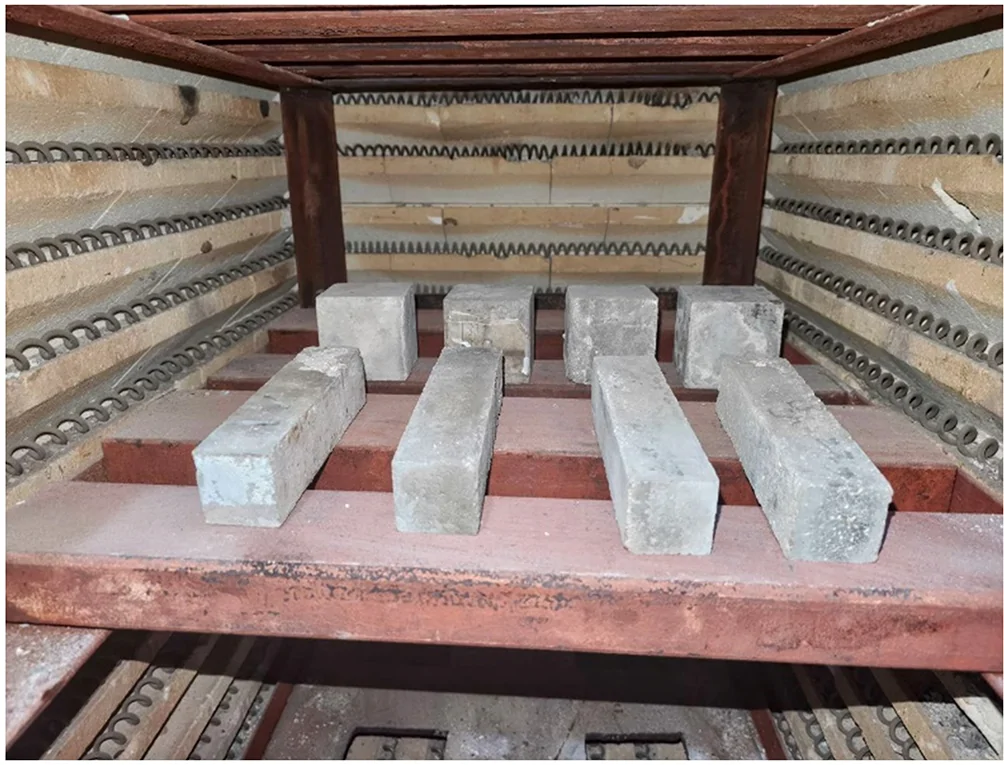
The process of applying high temperatures to specimens.

**Figure 3.**
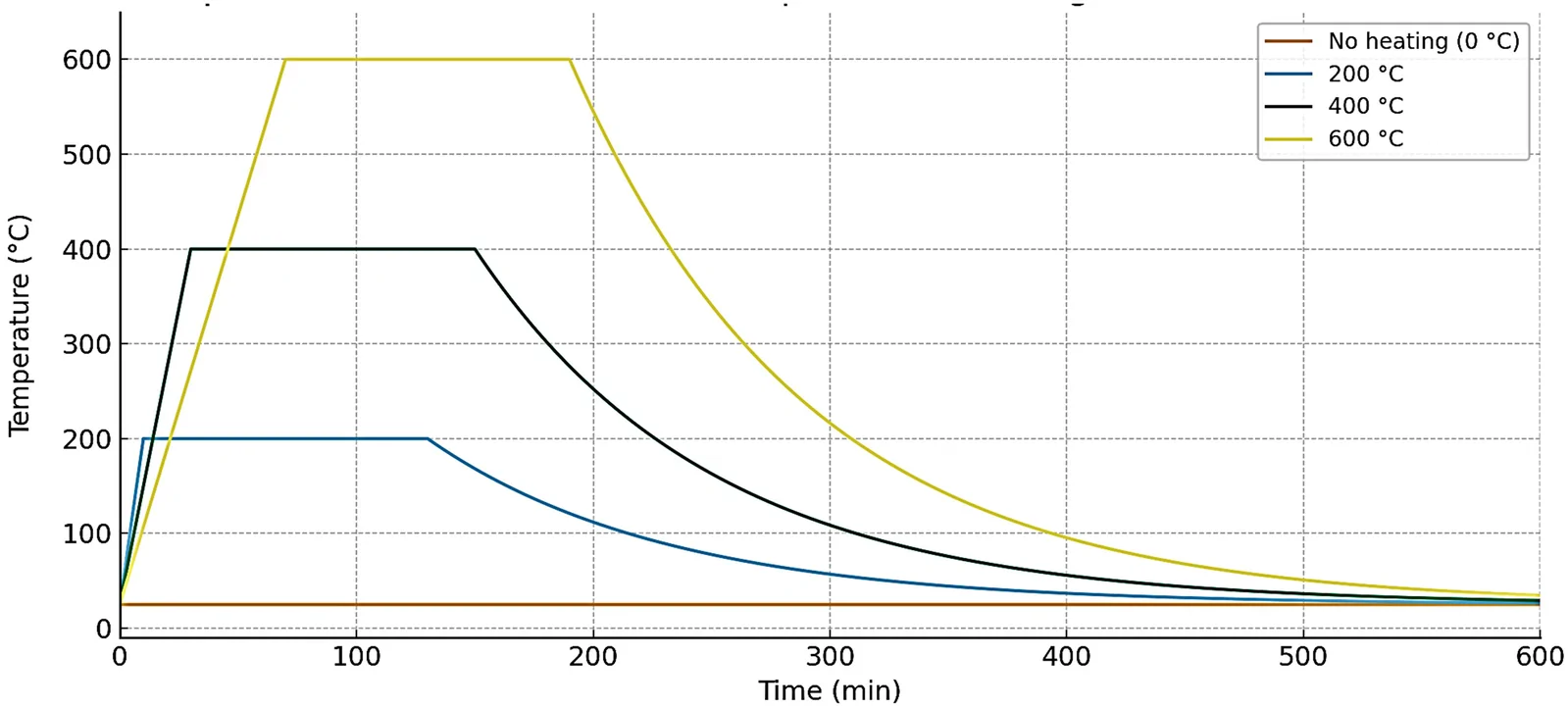
The temperatures profiles.

**Table 2.** Summary of specimen dimensions, testing standards, and number of specimens used in the experimental program (three specimens were tested for each mixture at each temperature level).

Test	Specimen size	No. of specimens	Test standard
Flow table	Mortar cone	3	ASTM C1437
Density	50 × 50 × 50 mm cubes	3	ASTM C642
Compressive strength	50 × 50 × 50 mm cubes	3	ASTM C109
Flexural strength & load–deflection	50 × 50 × 160 mm prisms	3	ASTM C348
UPV	50 × 50 × 160 mm prisms	3	ASTM C597

## 3. Results and Discussion

### 3.1 Slump flow

The data clearly shows a decrease in the workability of the mortar as the Polypropylene (PP) fiber content increases, see Figure 4. The flow diameter a measure of workability drops from 185 mm for the control mix to 125 mm 1.5% PP fibers.

**Figure 4.**
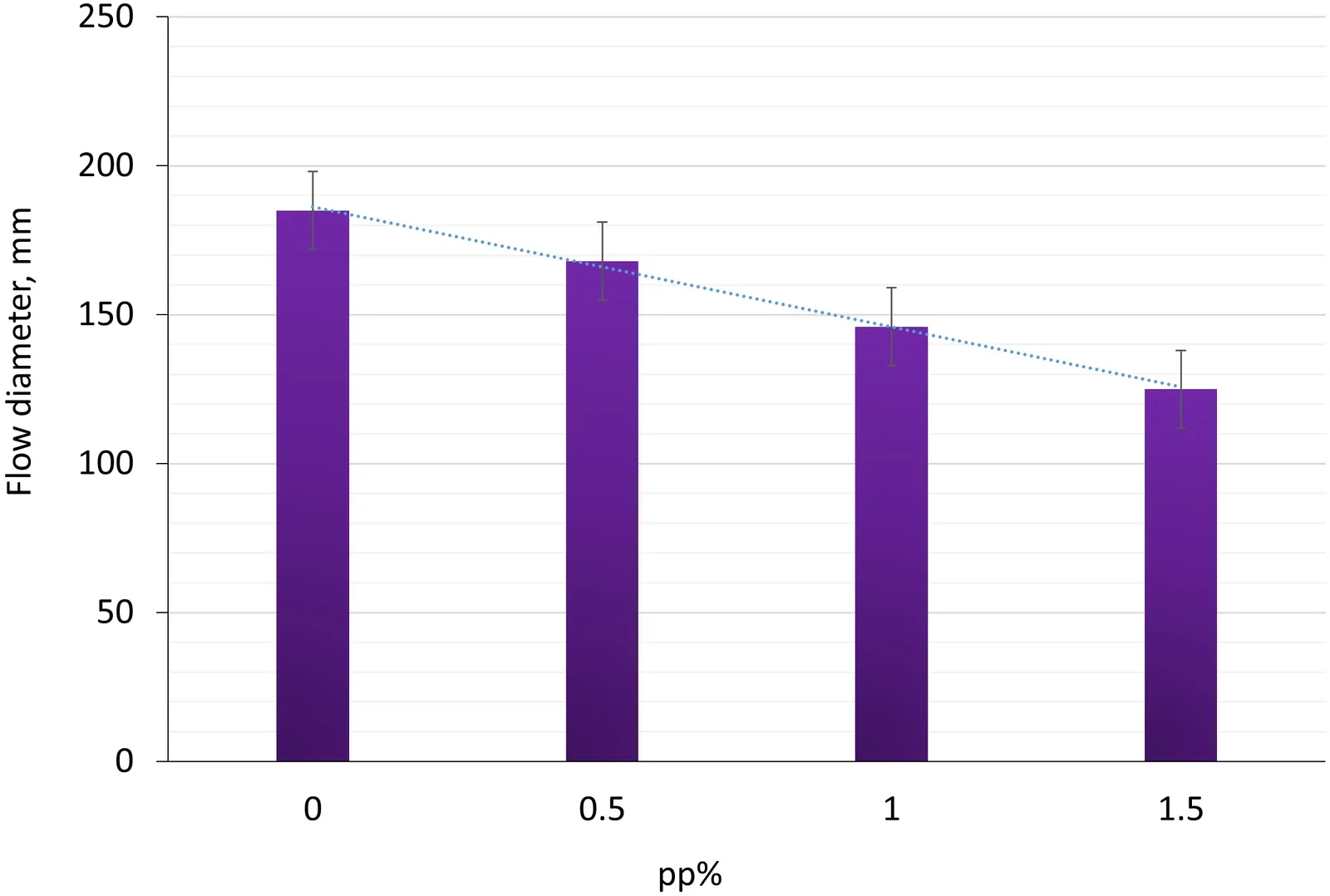
The flow diameter vs. pp%.

The flow diameter reduction in this study about 32.4%, which is nearly identical to the 25–35% reduction documented by Jawad and Al-Haydari ^27^ and Kumar et al. ^28^ confirming that workability reduction is a predictable consequence of increased PP fiber content.

The addition of a large number of fine PP fibers significantly increases the total surface area within the mix. Since the water/cement ratio and superplasticizer dosage are kept constant, the available free water (the water lubricating the particles) is reduced, leading to a drier, stiffer mix and lower workability. Also, the dispersed fibers create an internal network or “skeletal structure” within the mortar. This network physically interlocks the cement and sand particles, hindering their relative movement and sliding results in increased internal friction, higher surface area, and formation of a fiber network that restricts particle movement. Keeping the superplasticizer dosage constant allowed the study to realistically capture the practical consequences of increasing fiber content on both workability and mechanical performance. All specimens were carefully compacted in molds and prepared under identical casting and curing conditions to minimize the influence of consolidation variability on compressive strength results.

### 3.2 Density

Results are shown in Figure 5 and Table 3. As illustrated in Figure 5, the density of all mortar mixtures decreased progressively with the increase in temperature. This decline can be attributed to the evaporation of physically and chemically bound water, the decomposition of hydration products, and the development of internal microcracks within the matrix. At room temperature (25°C), the density values ranged between 2189 and 2293 kg/m ^3^ depending on the fiber content.

**Figure 5.**
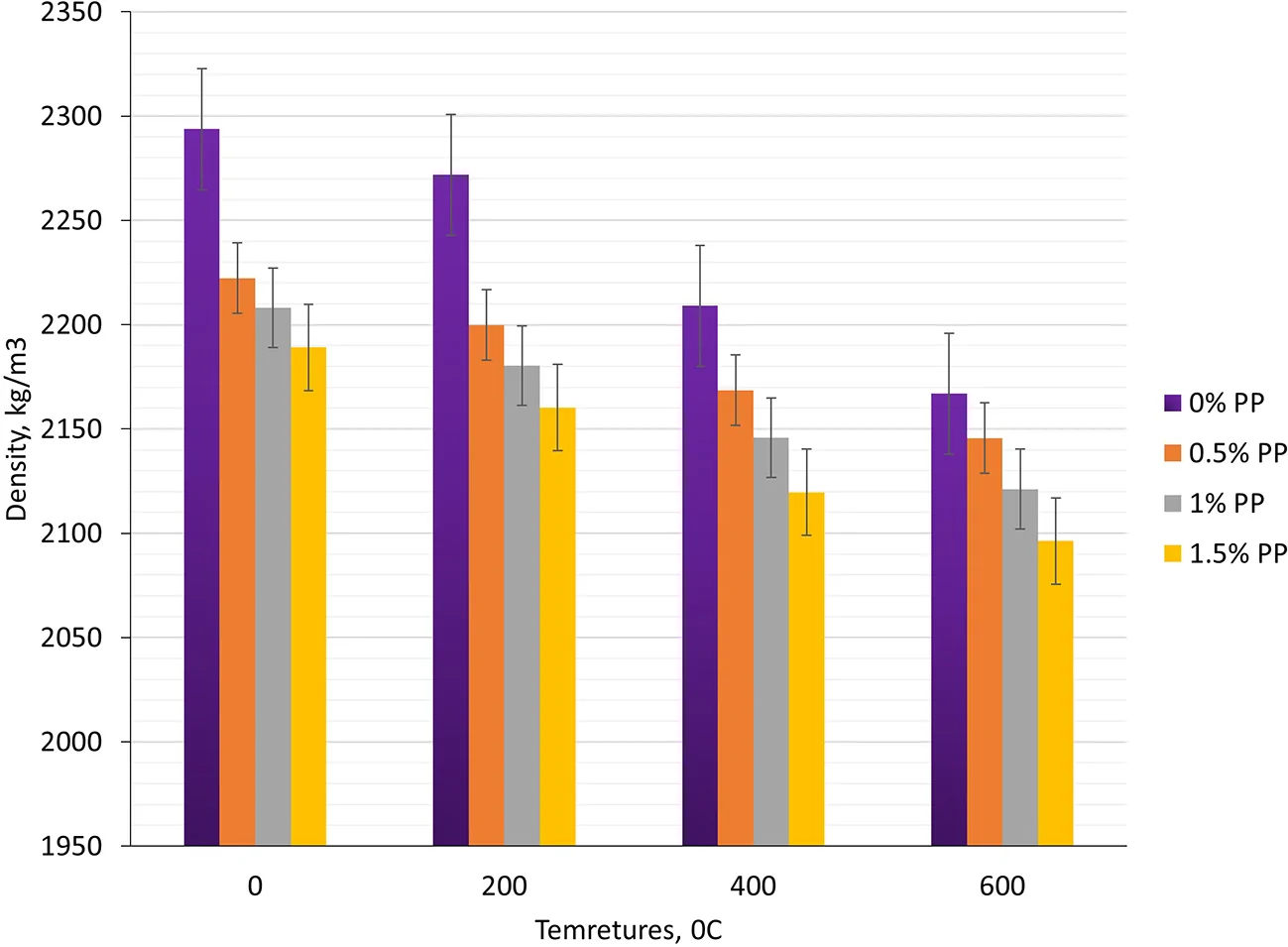
The density vs. pp% for different temperatures level.

**Table 3.** Dry density and residual density percentage at various pp% and temperature levels.

PP fiber content (%)	Temperature (°C)	Dry Density (kg/m ^3^)	Residual %
0.0	0	2293.74	100.0
	200	2271.87	99.0
	400	2209.05	96.3
	600	2166.91	94.5
0.5	0	2222.33	100.0
	200	2199.84	99.0
	400	2168.55	97.6
	600	2145.62	96.5
1.0	0	2208.12	100.0
	200	2180.41	98.7
	400	2145.77	97.2
	600	2121.18	96.0
1.5	0	2189.08	100.0
	200	2160.30	98.7
	400	2119.63	96.8
	600	2096.32	95.8

In comparison to the reference condition, the densities decreased by roughly 5–6% when the temperature rose to 600°C. Xiao and König ^29^ reported similar low density loss, suggesting that strength degradation is dominated by microstructural degeneration rather than bulk mass loss. They found 5–7% density loss for concrete at 600°C. Because PP fibers have a lower specific gravity than cementitious components, their incorporation somewhat decreased the initial density. At higher temperatures, though, the variations between the combinations lost some of their significance.

It can be observed from Table 3 that all mixes maintained over 94% of their original density up to 600°C. The control mix shows a slightly sharper reduction, reaching 94.5% retention, whereas the mixes with PP fibers residual strength was between 95.8% and 96.5% compared to control one. This improvement in density retention with the inclusion of PP fibers can be related to the fibermelting mechanism, which forms micro-channels allowing vapor escape and minimizing internal pressure buildup. Consequently, this mechanism mitigates explosive spalling and microcrack propagation, resulting in slightly better dimensional and mass stability under thermal exposure. Overall, the results suggest that adding PP fibers enhances the mortar’s ability to maintain its compactness and resist degradation at elevated temperatures.

### 3.3 Compressive strength

The Figures 6, 7 and Table 4 reports compressive strength for mixtures with 0, 0.5, 1.0 and 1.5% PP fibers after exposure to 0, 200, 400 and 600°C with Residual Strength (%).

**Figure 6.**
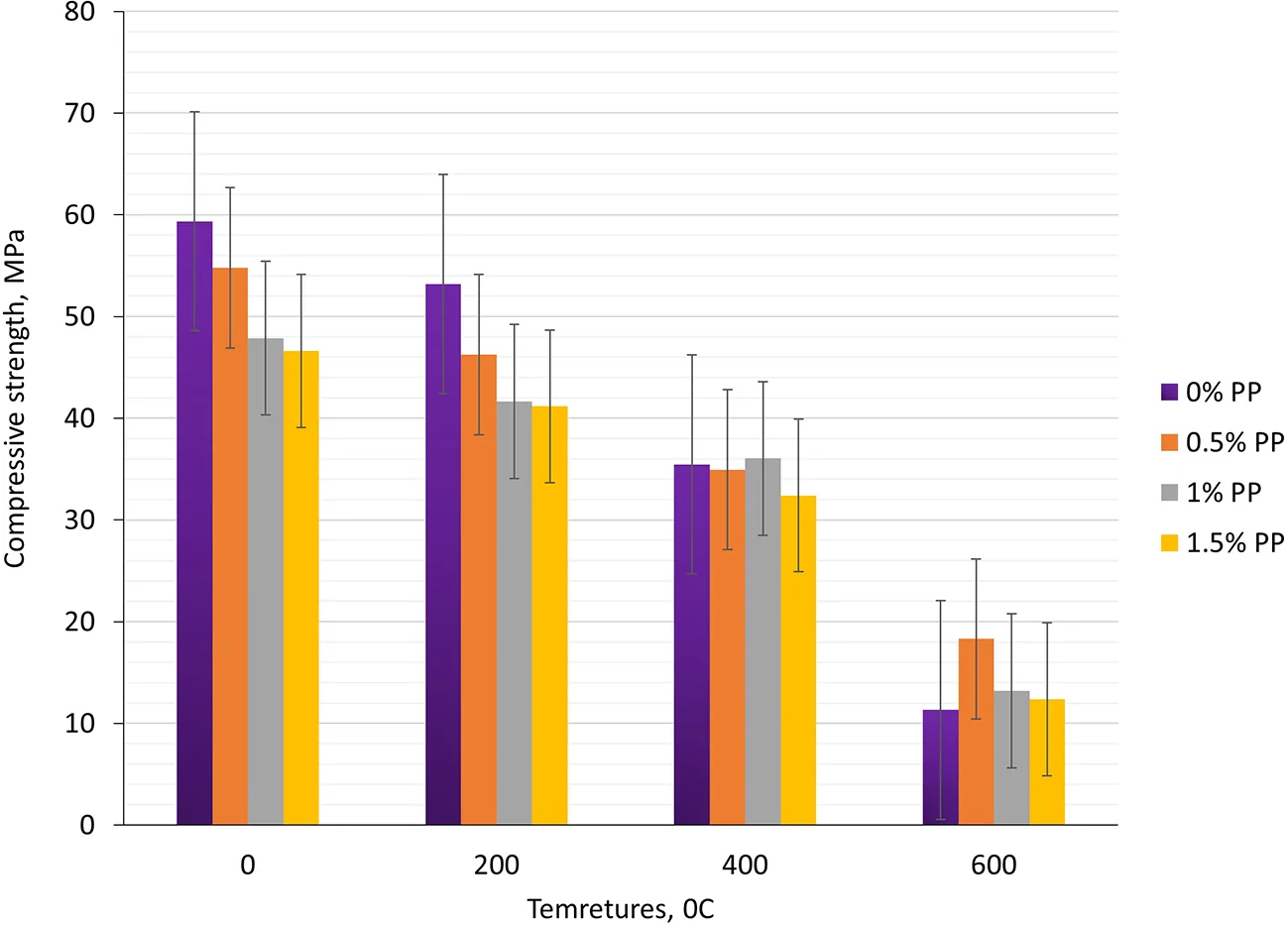
Compressive strength vs. temperature for different PP fiber contents.

**Figure 7.**
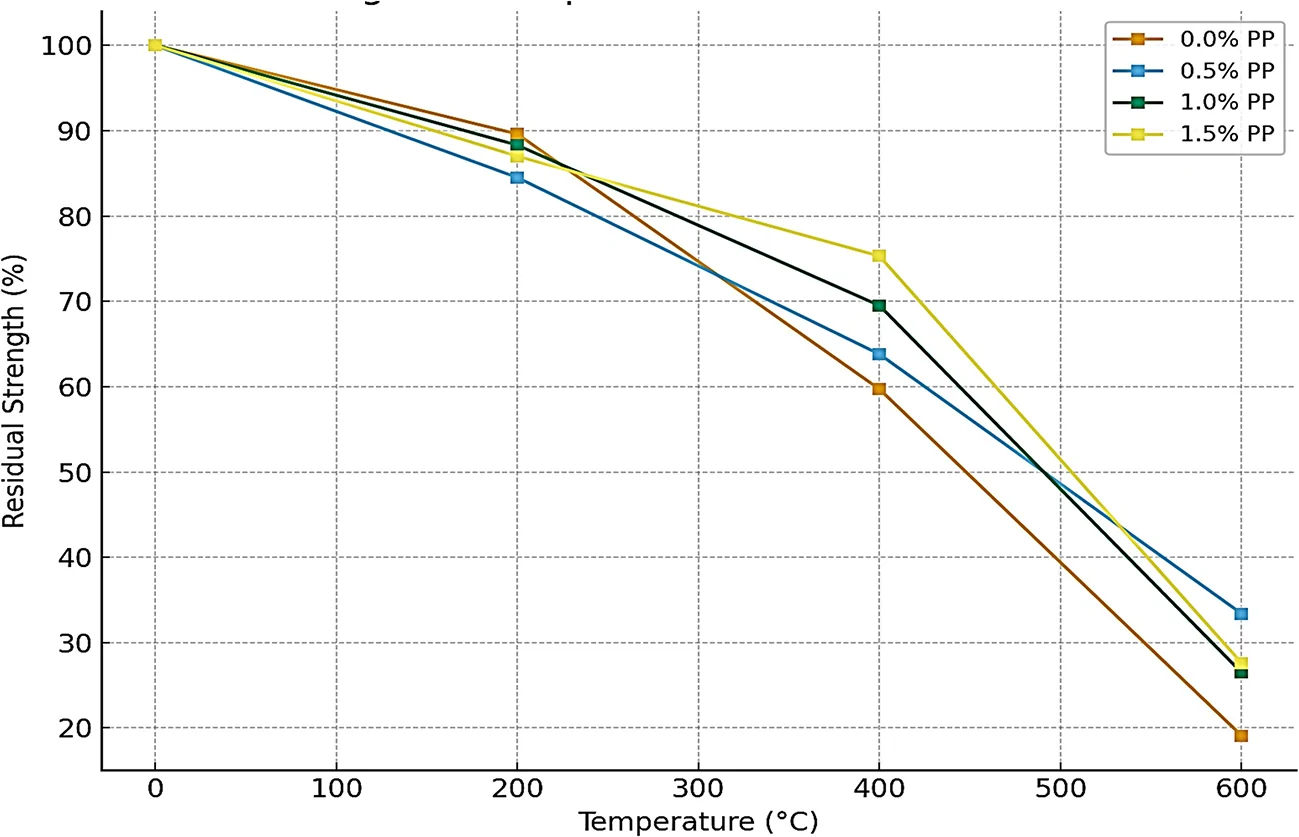
Residual strength vs. temperature for various PP fiber contents.

**Table 4.** The compressive and residual strength% for different pp% at different temperatures.

PP fiber content (%)	Temperature (°C)	Compressive strength (MPa)	Residual strength (%)
0.0	0	59.36	100.0
	200	53.19	89.6
	400	35.46	59.7
	600	11.32	19.1
0.5	0	54.77	100.0
	200	46.26	84.5
	400	34.95	63.8
	600	18.30	**33.4**
1.0	0	47.88	100.0
	200	41.65	87.0
	400	36.04	75.3
	600	13.21	27.6
1.5	0	46.61	100.0
	200	41.16	88.3
	400	32.41	69.5
	600	12.36	26.5

As shown in Figure 6, the compressive strength of all mortar specimens decreased progressively with increasing temperature, demonstrating the thermal degradation of the cementitious matrix. PP fibers slightly reduce compressive strength at ambient temperature due to increased porosity, their value lies in high-temperature performance, not initial strength. Upon heating, the fibers melt to produce pressure-relief channels that reduce spalling and limit microcracking. As a result, fiber-reinforced mortars retain much higher residual strength and toughness than the control mix, demonstrating that the benefit of fibers is post-fire structural integrity, not compressive strength at room temperature. ^30^ At 200°C, the reduction in strength was moderate (approximately 10–15%), primarily due to the evaporation of free water and the initiation of microcracking caused by thermal expansion mismatch between the cement paste and aggregates. ^30, 31^ Beyond 400°C, a sharp decline in strength was observed, particularly for the control mixture, which residual only about 60% of its initial strength. This behavior corresponds to the decomposition of calcium silicate hydrate (C–S–H) gel and partial dehydration of calcium hydroxide (CH), both of which weaken the matrix cohesion. ^32, 33^

At 600°C, the control mixture retained only 19.1% of its original compressive strength. In contrast, the inclusion of polypropylene fibers improved the residual strength of mortar. The 0.5% PP mixture showed the highest residual strength retention (33.4%), followed by the 1.0% PP mixture (27.6%) and the 1.5% PP mixture (26.5%). These results indicate that moderate fiber contents are more effective in mitigating thermal damage and preserving the structural integrity of mortar after high-temperature exposure. The melting of PP fibers at 160–170°C creates micro-channels that release internal vapor pressure, preventing explosive spalling and internal damage and contributing to the enhanced performance of fiber-reinforced specimens. ^34, 35^ This process slows the propagation of thermal cracks and improves the matrix’s structural integrity.

Husem ^6^ found residual strength of 20–22% for mortar at 600°C, while Alvarez et al. ^15^ found 30–35% residual strength for PP-reinforced mortar. Shahriar et al. ^16^ found 32–36% residual strength at 600°C for PP-fiber concrete.

This increased residual strength is in line with results from earlier research that showed PP fibers enhance cementitious composites’ high temperature resistance and post-heating recovery through encouraging vapor release, lowering pore pressure, and lessening the likelihood of explosive spalling. ^36, 37^ Additionally, the distributed fibers maintain improved load transfer inside the composite by acting as crack arresters throughout both heating and cooling cycles. ^38^ Overall, the findings show that a moderate amount of PP fibers (about 0.5% to 1.0%) can effectively reduce heat damage and improve mortar’s mechanical recovery after fire. But because to poor dispersion or increased porosity, an excessively high fiber content may somewhat lower the initial compressive strength.

### 3.4 Load–displacement curves

Both the ultimate load-carrying capacity (Pmax) and the deflection at failure (Δ) are clearly impacted by the PP fiber content and exposure temperature, according to the findings of the flexural tests shown in Figure 8 and Table 5. Flexural strength and ductility were significantly reduced (Δ dropped from 0.0600 mm to 0.0068 mm) in the fiber-free reference mix, which showed a steady decrease in Pmax from 2.80 kN at room temperature to 1.09 kN at 600°C. At all temperature settings, the addition of PP fibers improved the deformation capacity and load resistance. The blend with 0.5% PP fibers demonstrated better thermal stability by achieving a greater initial load capacity of 3.55 kN and maintaining 3.17 kN at 200°C.

**Figure 8.**
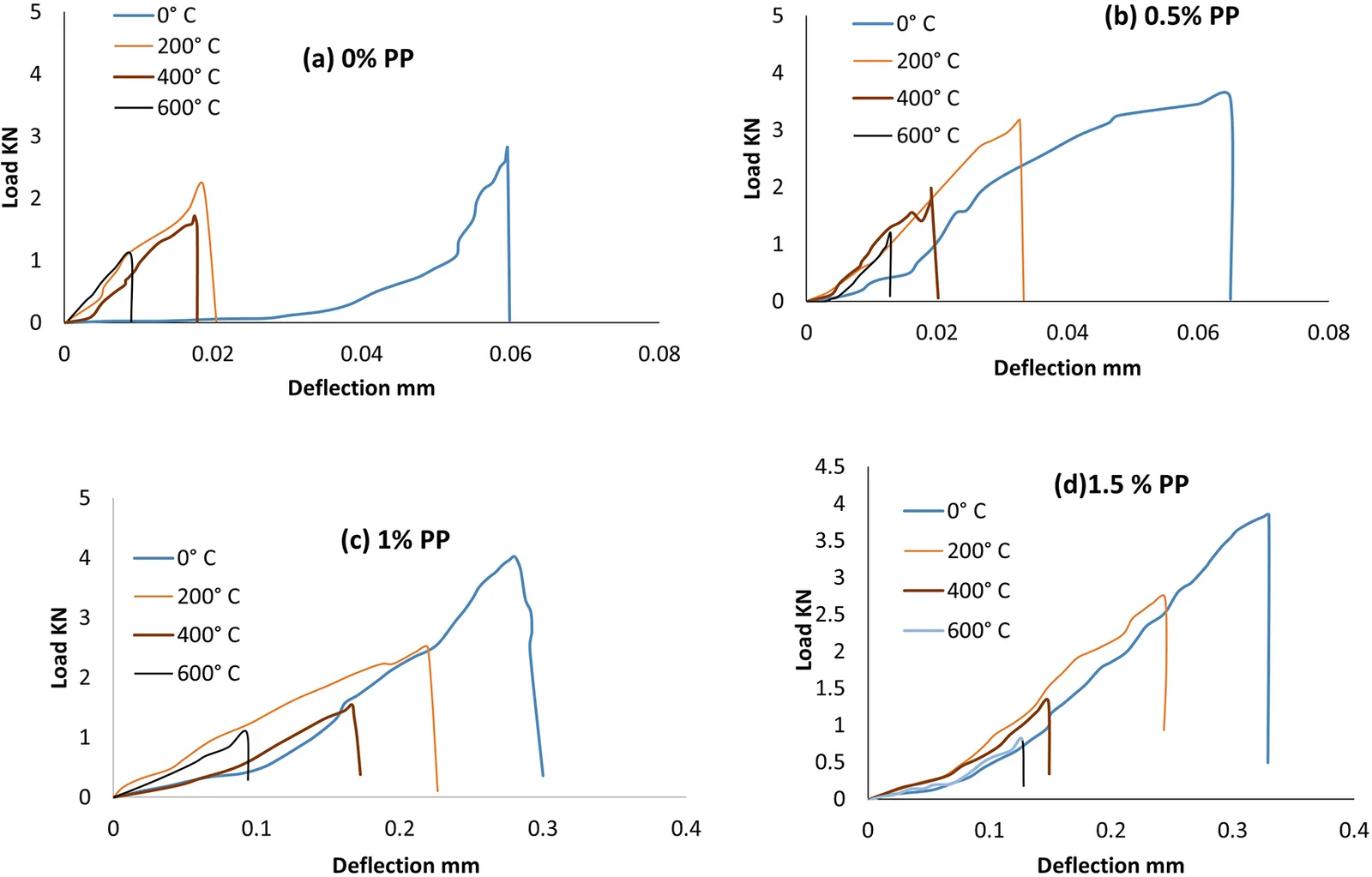
Load – Deflection vs. temperature for different PP fiber contents.

**Table 5.** The ultimate load and maximum recorded deflection at failure for different pp% for different temperatures level.

PP fiber content (%)	Temperature (°C)	Pmax (kN)	Δ at failure (mm)
0.0%	0	2.800	0.0600
	200	2.200	0.0205
	400	1.529	0.0180
	600	1.090	0.0068
0.5%	0	3.550	0.0650
	200	3.173	0.0330
	400	1.970	0.0600
	600	1.180	0.0130
1.0%	0	4.020	0.2800
	200	2.492	0.3000
	400	1.550	0.1660
	600	1.0952	0.0920
1.5%	0	3.820	0.3290
	200	2.730	0.2440
	400	1.325	0.1480
	600	0.857	0.1170

Despite fiber softening, this mix maintained a significant amount of ductility at 400°C (Δ = 0.0600 mm), suggesting efficient crack-bridging and stress redistribution. The deflection at ambient temperature (0.3 mm) and Δ at 200°C (0.2200 mm) for the fiber content to 1.0% resulted in a significant improvement in deformability compared to control mix, which mean improved post-cracking energy absorption due to fiber plasticization.

For fiber content (1.5% PP) a decreased in thermal resistance and load capacity have been notice, which may be result of pore development and fiber aggregation after melting. Due to the degradation of the cement matrix and the loss of fiber–matrix interaction, all mixtures exhibited a decrease in strength and ductility at high temperatures (400–600°C).

Polypropylene fibers soften and melt at approximately 160–170°C, forming micro-channels within the cementitious matrix that facilitate vapor release and reduce internal pore pressure during heating. The formation of these channels plays a significant role in mitigating explosive spalling in cementitious materials exposed to elevated temperatures. ^7, 38– 40^ At moderate temperatures (≤400°C), these fibers undergo partial softening, which promotes crack bridging and energy dissipation, thereby enhancing deflection capacity, as observed in the 0.5% and 1.0% PP mixes.

Higher temperatures (>400°C) cause the fibers to completely melt, which weakens the matrix–fiber interface and creates pores, lowering stiffness and load capacity. ^41^ The dehydration of C-S-H gel, the decarbonation of calcium carbonate, and the formation of microcracks are all responsible for the decrease in residual load capacity at 600°C that was seen across all fiber compositions. ^6– 8^ Despite this deterioration, PP fibers, especially when used at the optimal dosage of 0.5%, significantly postpone the loss of stiffness and maintain residual ductility by improving stress redistribution following cracking. A balanced fiber content can give synergistic increases in both thermal resistance and post-failure deformation capacity, according to similar findings reported by other researchers studying PP fiber-reinforced concretes exposed to heat. ^9– 11^

### 3.5 Flexural strength

The relationship between temperature and flexural strength (fr) for mortars with varying PP fiber concentrations is depicted in Figure 9. The σ of the control mix gradually decreased, going from 10.5 MPa at 25°C to 4.1 MPa at 600°C, or a 61% decrease. By achieving 13.3 MPa at 0.5% PP and 14.7 MPa at 1.0% PP, the addition of PP fibers greatly increased strength under ambient conditions, indicating a higher stress transfer through fiber bridging and decreased microcrack propagation. ^8, 33, 37^ All mixtures, however, demonstrated significant strength loss at 200°C and beyond as a result of matrix micro cracking, cement hydrate dehydration, and polymer melting. ^7, 32, 35^

**Figure 9.**
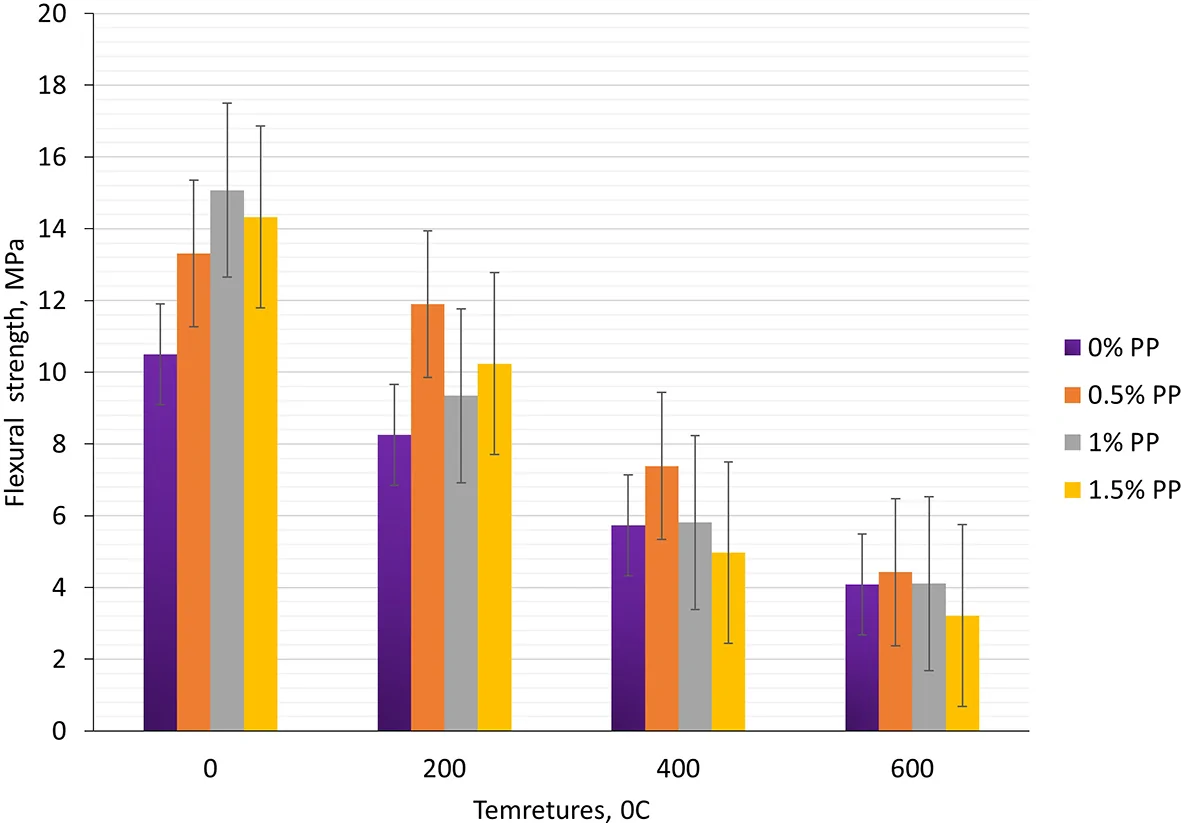
Flexural strength vs. temperature for different PP fiber contents.

The 0.5% PP mix retains about 42% of its original strength at 400°C, but the 1.0% and 1.5% PP mixes retain 38% and 35% of their initial strength, respectively. Fiber melting and pore formation caused further deterioration above 400°C. ^3, 42^ However, fiber-reinforced mixtures continued to perform better than unreinforced mortar, demonstrating the usefulness of PP in redistributing stress prior to melting. The percentage change in σ with temperature is shown in Table 6, which shows that the highest relative loss happens above 400°C. According to the trend, polymer combustion takes precedence over the crack-bridging action above a threshold temperature of around 350 to 400°C. ^43^ The area under the load-displacement curve, or estimated toughness, shows that the use of PP significantly improves post-peak energy absorption.

**Table 6.** Flexural strength vs. PP% at different temperatures.

PP fiber content (%)	Temperature (°C)	f _r_ (MPa)	% change
0.0%	0	10.5000	—
0.5%	0	13.3125	+26.83%
1.0%	0	15.0750	+43.57%
1.5%	0	14.3250	+36.43%
0.0%	200	8.2500	—
0.5%	200	11.8988	+44.25%
1.0%	200	9.3450	+13.27%
1.5%	200	10.2375	+24.09%
0.0%	400	5.7338	—
0.5%	400	7.3875	+28.85%
1.0%	400	5.8125	+1.37%
1.5%	400	4.9688	−13.34%
0.0%	600	4.0875	—
0.5%	600	4.4250	+8.26%
1.0%	600	4.1070	+0.48%
1.5%	600	3.2138	−21.38%

The flexural strength retention observed in the present study (55% for 0.5% PP and 38–42% for other mixes at 400°C) aligns well with the 30–45% range reported by Noumowé ^42^ and the 35–50% range noted by Li et al. ^43^ These quantitative similarities confirm that PP-reinforced mortar exhibits a similar degradation trend to PP-reinforced concrete under thermal loading.

### 3.6 Toughness

The flexural toughness has been calculated from area under load-displacement curves and the results illustrated in Figure 10 and Table 7. For these results, at ambient temperature, the flexural toughness rose from 0.052 N·mm/mm ^2^ for control specimen to 0.072 N·mm/mm ^2^ for specimens contain 0.5% PP, resulted in higher ductility and better deformation capacity. This improvement is in line with recent studies that demonstrate that lower dosages of PP encourage matrix–fiber synergy, but higher dosages may result in fiber aggregation and voids. ^44, 45^ Up to 1.0% PP addition often improves flexural performance and heat resistance at mild temperatures (≤400°C); however, structural pore expansion and polymer disintegration cause significant degradation above this range. ^37, 45^ At elevated temperatures, at 600°C, the 1.5% PP mix retained a residual toughness of 50 N·mm, which was 10 times higher than the control, indicating maintained post-cracking deformation capability. ^43^

**Figure 10.**
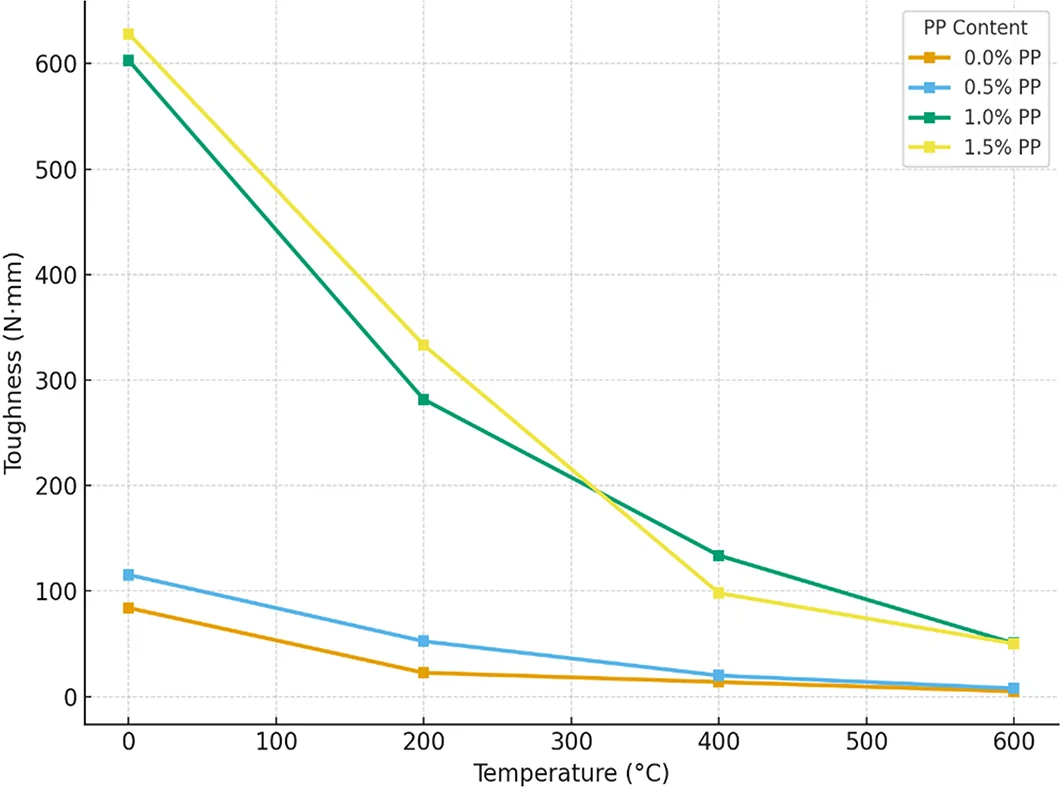
Flexural toughness vs. temperature for different PP fiber contents.

**Table 7.** Flexural toughness and percentage change relative to the control mix (0% PP) at each temperature

PP fiber content (%)	Temperature (°C)	Toughness (N·mm)	% change vs control
0.0	0	85	0%
0.5	0	115	+35.3%
1.0	0	600	+605.9%
1.5	0	630	+641.2%
0.0	200	25	0%
0.5	200	50	+100%
1.0	200	280	+1020%
1.5	200	335	+1240%
0.0	400	15	0%
0.5	400	20	+33.3%
1.0	400	135	+800%
1.5	400	100	+566.7%
0.0	600	5	0%
0.5	600	10	+100%
1.0	600	50	+900%
1.5	600	50	+900%

These results are in line with prior studies showing that post-peak energy dissipation in high-temperature concrete is greatly enhanced by small PP additions (0.5–1.0%). ^46^ While maintaining exceptional thermal stability, too much PP (>1.0%) may cause voids that compromise matrix integrity at room temperature. ^9, 43^ Overall, the toughness trends demonstrate that PP fibers are helpful in lowering heat-induced brittleness and enhancing residual performance by improving ductility and energy absorption under both normal and thermal stress settings.

### 3.7 Ultrasonic impulse velocity 

To provide additional insight into the internal damage and microstructural deterioration induced by elevated temperatures, Ultrasonic Pulse Velocity (UPV) measurements were conducted on all mortar mixtures. UPV is a widely used non-destructive testing (NDT) technique for evaluating internal defects, microcracking, and changes in the pore structure of cementitious materials. The results presented in Table 8 allow a more quantitative assessment of the internal degradation occurring in the mortar specimens after exposure to temperatures of 200, 400, and 600°C. According to the UPV classification criteria, the mortar mixtures exhibited excellent to good quality at ambient temperature, while exposure to elevated temperatures progressively reduced the internal quality of the specimens. At 600°C, most mixtures fall within the medium to poor quality range, indicating significant internal microstructural damage caused by thermal exposure. The reduction in UPV values with increasing polypropylene fiber content at ambient temperature does not necessarily indicate deterioration in mechanical performance. This behavior can be attributed to the introduction of additional interfaces and micro-voids within the mortar matrix due to fiber dispersion. These interfaces slightly reduce the continuity of the solid phase and consequently slow the propagation of ultrasonic waves. ^47^

**Table 8.** UPV results of mortar mixtures with different polypropylene fiber contents after exposure to elevated temperatures.

PP fiber content (%)	Temperature (°C)	UPV (km/s)	Change (%) vs control at same temperature	Concrete Quality
0.0	0	4.70	0.0	Excellent
0.5	0	4.50	−4.3	Good
1.0	0	4.20	−10.6	Good
1.5	0	4.00	−14.9	Good
0.0	200	4.30	0.0	Good
0.5	200	4.20	−2.3	Good
1.0	200	4.00	−7.0	Good
1.5	200	3.80	−11.6	Good
0.0	400	3.70	0.0	Good
0.5	400	3.60	−2.7	Good
1.0	400	3.40	−8.1	Medium
1.5	400	3.20	−13.5	Medium
0.0	600	3.00	0.0	Medium
0.5	600	2.90	−3.3	Poor
1.0	600	2.70	−10.0	Poor
1.5	600	2.50	−16.7	Poor

Despite this reduction in wave velocity, polypropylene fibers enhance the crack-bridging capability and post-cracking energy absorption of the mortar. Therefore, the decrease in UPV mainly reflects changes in the internal pore structure rather than a reduction in structural performance. Similar observations have been reported in previous studies on fiber-reinforced cementitious composites, where the presence of fibers increases ductility and toughness while slightly reducing ultrasonic pulse velocity due to increased heterogeneity in the matrix.

## 4. Conclusion

This study examined the mechanical performance and thermal resistance of mortar containing 0–1.5% PP fibers exposed to temperatures up to 600°C. The following conclusions have been drawn based on results:

1. Incorporating PP fibers decreased flow diameter from 185 mm (0% PP) to 125 mm (1.5% PP), representing a 32.4% reduction, primarily due to increased internal friction and surface area.
2. All mixtures retained above 94% of their original density at 600°C. The control mix kept 94.5%, whereas PP fiber mixes retained slightly higher values (96.0–96.5%), confirming that melted fibers help limit microcrack propagation.
3. At ambient temperature, compressive strength decreased with fiber inclusion (from 59.36 MPa for the control to 46.61–54.77 MPa for PP mixes.
4. At 600°C, however, PP fibers improved residual strength, for control mix was 19.1% residual strength while for 0.5% PP was 33.4% residual strength (75% improvement over control), 26.5% for 1.0% PP, and 27.6% for 1.5% PP.
5. At ambient temperature, flexural strength increased significantly with PP fibers.
6. After exposure to 600°C, fiber-reinforced mixes kept 0.5–8.3% more flexural strength compared to the control.
7. Flexural toughness improved markedly due to fiber bridging. At 600°C, the 1.5% PP mix retained toughness values nearly 10 times higher than the control, confirming substantial enhancement in post-cracking energy absorption.
8. Based on results of all tested made in this study, 0.5% PP fibers provided the best balance between initial performance and high-temperature residual behavior, offering the highest combined improvement in residual compressive and flexural, and ductility.

The present study is limited to a single PP fiber geometry and one mortar mix design (w/c = 0.45; cement:sand = 1:3) with a maximum exposure temperature of 600°C under furnace cooling conditions. In addition, long-term durability aspects after thermal exposure were not examined. Future research will investigate alternative fiber systems (e.g., basalt, steel, or hybrid fibers), higher fire temperatures (800–1000°C), different cooling regimes, and microstructural characterization techniques (e.g., SEM and porosity analysis) to provide deeper insight into thermal damage mechanisms.

## Data Availability

The datasets supporting the finding of this study are openly available in Zenodo: Data Manuscript: Performance of Polypropylene Fiber-Reinforced Mortar Exposed to Elevated Temperatures repository at https://doi.org/10.5281/zenodo.18056598. ^48^ This project contains the following data:
•
The data avialability file.xlsb Data are available under the terms of the Creative Commons Attribution 4.0 International license (CC-BY 4.0).
