# Supplementary material for: Performance of Polypropylene Fiber-Reinforced Mortar Exposed to Elevated Temperatures
Source: F1000Res. 2026 Mar 19;15:30. Originally published 2026 Jan 8. [Version 2] doi: 10.12688/f1000research.175541.2 (PMC13554995; doi:10.12688/f1000research.175541.2)
Supplement: Peer review history

# Reviewer report: Reviewer response for version 2

**DOI:** 10.5256/f1000research.197465.r497918

**Published:** 2026-07-28

**Authors:** Aktham Hatem Qasim

**Affiliations:** 1 University of Fallujah, Fallujah, Iraq

1. Subsection 2.1 (The description of the superplasticizer is insufficient). The authors should specify the commercial product, manufacturer, chemical base (e.g., polycarboxylate ether), etc.

 2. Add fig. of PP fiber used in the current study>

 3. Table 1 (Better move the PP fiber column to the end of the table and remove the percentage symbol from its values. In addition, remove the percentage symbol from the Superplasticizer entries, as the unit is already indicated in the column heading.)

 4.Add a failure mode for the flexural strength test.

 5. Subsectoin 3.1, The addition of a large number of fine PP fibers....... (Better change to volume fraction or  fiber content).

 6.Subsection 3.2 (Please add appropriate references to substantiate this attribution.)

 7.Please correct the temperature unit on the x-axis in Figures 5 and 6 from "0C" to the standard notation "°C".

 8. Subsection 3.3 (33–28%, respectively) (Please ensure consistency between the numerical values reported in the text and those presented in Table 3.)

 In my opinion, the manuscript is scientifically valid in its current form. The comments provided in my review are intended as **minor, non-essential suggestions** to improve the clarity and presentation of the manuscript, and they do not affect the scientific validity of the study or its conclusions.

Is the work clearly and accurately presented and does it cite the current literature?

Partly

If applicable, is the statistical analysis and its interpretation appropriate?

Not applicable

Are all the source data underlying the results available to ensure full reproducibility?

Yes

Is the study design appropriate and is the work technically sound?

Yes

Are the conclusions drawn adequately supported by the results?

Yes

Are sufficient details of methods and analysis provided to allow replication by others?

Yes

Reviewer Expertise:

Construction Materials, Materials, Green Concrete, Composite Concrete, Cementitious Materials, Fiber-Reinforced Concrete, Sustainable Construction Materials, Concrete Durability, High-Performance Concrete, Structural Materials.

I confirm that I have read this submission and believe that I have an appropriate level of expertise to confirm that it is of an acceptable scientific standard.

# Reviewer report: Reviewer response for version 2

**DOI:** 10.5256/f1000research.197465.r469065

**Published:** 2026-03-28

**Authors:** öznur Biricik

**Affiliations:** 1 Bursa Uludag University, Nilüfer-Bursa, Turkey

The revisions are sufficient for **indexing .**

Is the work clearly and accurately presented and does it cite the current literature?

Partly

If applicable, is the statistical analysis and its interpretation appropriate?

Not applicable

Are all the source data underlying the results available to ensure full reproducibility?

Yes

Is the study design appropriate and is the work technically sound?

Yes

Are the conclusions drawn adequately supported by the results?

Yes

Are sufficient details of methods and analysis provided to allow replication by others?

Yes

Reviewer Expertise:

Concrete technology

I confirm that I have read this submission and believe that I have an appropriate level of expertise to confirm that it is of an acceptable scientific standard.

# Reviewer report: Reviewer response for version 1

**DOI:** 10.5256/f1000research.193533.r461087

**Published:** 2026-03-02

**Authors:** Kaveh Roushan

**Affiliations:** 1 University of Missouri, Columbia, USA

**General Evaluation**

 This study examines the mechanical and thermal properties of cement mortar reinforced with polypropylene (PP) fibers at volume fractions of 0%, 0.5%, 1.0%, and 1.5%, subjected to temperatures of 200°C, 400°C, and 600°C. It is highly relevant for fire-resistant building applications. A key strength is its focus on mortar specifically, clearly distinguishing it from concrete in terms of pore structure, thermal response, and cracking behavior, thus filling an important research gap. Identifying 0.5% PP fiber as the optimal balance between initial strength and residual fire performance offers a practical takeaway. Additionally, making the raw dataset available on Zenodo enhances transparency and reproducibility.

**Specific Comments**

**1. Absence of Microstructural Evidence: **The paper's main mechanistic claim — that melting PP fibers form micro-channels that reduce internal vapor pressure and prevent explosive spalling — is reiterated throughout the results and discussion but lacks direct evidence. No SEM images are included, making this key assertion speculative. To support this, SEM images of fractured mortar cross-sections after exposure to 200°C, 400°C, and 600°C should be provided for both the control and fiber-reinforced mixes, with annotations highlighting micro-channel formation, fiber remnants, the fiber–matrix interface, and void distribution. Additionally, quantitative porosity measurements via Mercury Intrusion Porosimetry (MIP) or Ultrasonic Pulse Velocity (UPV) testing would strengthen the existing density and strength data.

**2. Superplasticizer Characterization and Workability Methodology: **The manuscript mentions '1% superplasticizer type F' but does not specify the manufacturer, product name, code, or chemical base, which are necessary for reproducibility. More importantly, the superplasticizer dosage was kept constant across all mixes while PP fiber content varied from 0% to 1.5%. This caused a 32.4% reduction in flow diameter in the highest fiber mix, potentially affecting casting quality and confounding the interpretation of compressive strength at higher fiber levels. The lower strength might partly result from reduced workability rather than fiber effects alone. The authors should either (a) modify the superplasticizer dosage for each mix to ensure consistent workability before casting strength tests, or (b) clearly state this as a study limitation and estimate its possible influence on the results.

**3. Missing Specimen Replication Details and Flexural Testing Setup: **The methodology lacks details on the number of specimens used for density measurements, and the overall specimen count for each property–temperature–fiber combination is not summarized. A table should be included, listing specimen counts, dimensions, and related test standards for each measured property. Furthermore, Section 2.2 does not clarify whether flexural testing was conducted under displacement or load control, nor does it specify the crosshead speed (mm/min) or the model and capacity of the testing machine. Since toughness values are obtained from load–deflection curves, knowing the testing rate is essential; without this, the toughness data cannot be independently verified or reproduced.

**4. PP Fiber Type and Internal Melting Temperature Inconsistency: **The fiber type (monofilament or fibrillated/multifilament) and the manufacturer or product name are not specified. These details affect fiber–matrix bonding, dispersion, and thermal properties, and should be included as standard information. Additionally, there is an inconsistency regarding the melting temperature: the abstract and Section 2.1 report a melting point of 160–170°C, whereas Section 3.4 states that PP fibers 'melt near 320–340°C'. This discrepancy needs clarification and resolution. The 160–170°C range corresponds to the standard melting point of isotactic polypropylene, while the higher temperature likely refers to a different thermal or kinetic condition. Consistent use of the correct values throughout the document is essential manuscript.

**5. Curing Conditions: **The manuscript states that curing occurred 'in water at 20 ± 2°C until 28 days old," but it does not clarify whether the water was lime-saturated, which is the standard method to prevent calcium leaching from mortar and influences hydration products and final strength. Additionally, it is not mentioned if the curing water was renewed regularly. These details should be included.

**6. Novelty Justification and Literature Differentiation: **Although the mortar-vs-concrete distinction is presented as the novelty claim, the Introduction lacks a detailed mechanistic comparison to fully justify why this difference merits separate investigation. The literature should be cited to discuss the specific quantitative differences in paste-to-aggregate ratio, pore-size distribution, and thermal cracking mode between mortar and concrete. Additionally, in Sections 3.3 and 3.5, where results are compared with previous studies, it should be clearer whether those studies examined mortar or concrete. Each comparison ought to specify the material system used in the cited work and emphasize the mortar-specific findings of the current study.

**7. Internal Inconsistency in Compressive Strength Reporting: **In Section 3.3, it states that mortars reinforced with PP fiber 'maintain a comparatively higher strength (33–28%, respectively)' for the 0.5% and 1.5% PP mixes at 600°C. However, Table 3 shows the 1.0% PP mix retains 27.6%, and the 1.5% PP mix retains 26.5%. The 1.5% mix is not the pair being described. The text should be revised to accurately reflect the tabulated values for each mix separately and organize the performance ranking clearly.

**8. Dedicated Limitations and Future Work Section: **The manuscript lacks a 'Limitations and Future Work' section, which is a notable omission. The following limitations should be explicitly recognized: (a) only one PP fiber geometry was tested; comparing it with basalt, steel, or hybrid systems would enhance the applicability; (b) only a single mortar mix design (w/c = 0.45, 1:3 cement to sand) was studied; (c) the highest temperature tested, 600°C, does not encompass more extreme fire scenarios reaching 800–1000°C; (d) long-term durability aspects such as carbonation, chloride resistance, and creep after thermal exposure were not evaluated; and (e) the influence of different cooling methods (furnace cooling versus water quenching) on residual properties was not addressed, although this is crucial for post-fire structural assessments.

**9. Minor Typographical and Presentation Errors: **Several typographical and presentation errors need correction. The axis label 'Temretures, 0C' in Figures 5, 6, and 9 should be amended to 'Temperature (°C)'. The term 'formulation' in Section 3.5 should be changed to 'formation' ('pore formation'). The phrase 'control mia' in Section 3.4 should be corrected to 'control mix'. The label 'pp%' in Figure 4 should be standardized as 'PP fiber content (%)'. Lastly, Figure 10 (toughness) would be improved by adding a supplementary data table similar to Tables 2–5, enabling readers to easily access the exact numerical toughness values.

Is the work clearly and accurately presented and does it cite the current literature?

Partly

If applicable, is the statistical analysis and its interpretation appropriate?

Not applicable

Are all the source data underlying the results available to ensure full reproducibility?

Yes

Is the study design appropriate and is the work technically sound?

Partly

Are the conclusions drawn adequately supported by the results?

Partly

Are sufficient details of methods and analysis provided to allow replication by others?

Partly

Reviewer Expertise:

Soil stabilization, soil improvement, fiber reinforcement, novel stabilizer, durability cycles, energy engineering, geothermal energy, shallow geothermal energy, energy piles, energy micropiles, energy helical piles, numerical simulation, FEM.

I confirm that I have read this submission and believe that I have an appropriate level of expertise to confirm that it is of an acceptable scientific standard, however I have significant reservations, as outlined above.

# Response

**Published:** 2026-03-05

**Authors:** Sheelan Hama

**Affiliations:** Civil Engineering, University of Anbar, Ramadi, Al Anbar Governorate, Iraq

Specific Comments

**1. Absence of Microstructural Evidence: **The paper's main mechanistic claim — that melting PP fibers form micro-channels that reduce internal vapor pressure and prevent explosive spalling — is reiterated throughout the results and discussion but lacks direct evidence. No SEM images are included, making this key assertion speculative. To support this, SEM images of fractured mortar cross-sections after exposure to 200°C, 400°C, and 600°C should be provided for both the control and fiber-reinforced mixes, with annotations highlighting micro-channel formation, fiber remnants, the fiber–matrix interface, and void distribution. Additionally, quantitative porosity measurements via Mercury Intrusion Porosimetry (MIP) or Ultrasonic Pulse Velocity (UPV) testing would strengthen the existing density and strength data.

**Response to reviewer comment:** While Scanning Electron Microscopy (SEM) observations would indeed provide direct microstructural visualization, SEM analysis was not performed in the present experimental program due to laboratory limitations. Nevertheless, in response to the reviewer’s suggestion, Ultrasonic Pulse Velocity (UPV) testing has been conducted and incorporated into the revised manuscript to provide additional quantitative insight into the internal damage and microstructural deterioration of the mortar specimens after thermal exposure.

 A new subsection entitled “3.7 Ultrasonic Pulse Velocity (UPV) Analysis” has been added to the Results and Discussion section. The UPV results show a progressive reduction in wave velocity with increasing temperature and fiber content, which indicates the development of internal microcracks, pore expansion, and increased internal voids within the mortar matrix. These observations are consistent with the reductions observed in density and mechanical strength.

 The authors acknowledge that future work will include detailed microstructural characterization using SEM and pore structure analysis techniques such as Mercury Intrusion Porosimetry (MIP) to further investigate the micro-channel formation and pore evolution in heated mortar.

 2. **Superplasticizer Characterization and Workability Methodology: **The manuscript mentions '1% superplasticizer type F' but does not specify the manufacturer, product name, code, or chemical base, which are necessary for reproducibility. More importantly, the superplasticizer dosage was kept constant across all mixes while PP fiber content varied from 0% to 1.5%. This caused a 32.4% reduction in flow diameter in the highest fiber mix, potentially affecting casting quality and confounding the interpretation of compressive strength at higher fiber levels. The lower strength might partly result from reduced workability rather than fiber effects alone. The authors should either (a) modify the superplasticizer dosage for each mix to ensure consistent workability before casting strength tests, or (b) clearly state this as a study limitation and estimate its possible influence on the results.

**Response to reviewer comment:** The Materials section has been revised to clearly describe the type and technical properties of the superplasticizer used in this study. The manuscript now specifies that a polycarboxylate-based high-range water-reducing admixture, Sika ViscoCrete-180 GS, conforming to ASTM C494 Type F, was used. Additional information regarding its specific gravity (1.07), pH range (4–6), color (light brownish), and dosage (1% by weight of cement) has been added to improve the clarity and reproducibility of the experimental methodology.

 “A polycarboxylate-based high-range water-reducing admixture (HRWR), Sika ViscoCrete-180 GS, conforming to ASTM C494 Type F, was used to improve the workability of the mortar mixtures. The superplasticizer has a specific gravity of approximately 1.07, pH value ranging from 4 to 6, and a light brownish liquid appearance. The admixture is based on modified polycarboxylate ether technology and was added at a constant dosage of 1% by weight of cement for all mixtures to maintain comparable workability conditions while isolating the effect of polypropylene fiber content on the fresh and hardened properties of the mortar.”

 3. **Missing Specimen Replication Details and Flexural Testing Setup:** The methodology lacks details on the number of specimens used for density measurements, and the overall specimen count for each property–temperature–fiber combination is not summarized. A table should be included, listing specimen counts, dimensions, and related test standards for each measured property. Furthermore, Section 2.2 does not clarify whether flexural testing was conducted under displacement or load control, nor does it specify the crosshead speed (mm/min) or the model and capacity of the testing machine. Since toughness values are obtained from load–deflection curves, knowing the testing rate is essential; without this, the toughness data cannot be independently verified or reproduced.

**Response to reviewer comment: **A new summary table has also been added to the manuscript to clearly present the specimen count, specimen size, and relevant test standards used for density, compressive strength, flexural strength, and UPV measurements, see Table 2.

 Also, Section 2.2 has been expanded to clarify the flexural testing procedure and loading conditions. The manuscript now specifies that the flexural tests were performed using a three-point bending configuration on prism specimens (50 × 50 × 160 mm) in accordance with ASTM C348. The tests were conducted using a universal testing machine with a capacity of 300 kN, and the loading was applied under load control at a constant rate of 50 ± 10 N/s, as specified in the standard. During testing, the load–deflection response was recorded to evaluate post-cracking behavior and flexural toughness.

 4. **PP Fiber Type and Internal Melting Temperature Inconsistency:** The fiber type (monofilament or fibrillated/multifilament) and the manufacturer or product name are not specified. These details affect fiber–matrix bonding, dispersion, and thermal properties, and should be included as standard information. Additionally, there is an inconsistency regarding the melting temperature: the abstract and Section 2.1 report a melting point of 160–170°C, whereas Section 3.4 states that PP fibers 'melt near 320–340°C'. This discrepancy needs clarification and resolution. The 160–170°C range corresponds to the standard melting point of isotactic polypropylene, while the higher temperature likely refers to a different thermal or kinetic condition. Consistent use of the correct values throughout the document is essential manuscript.

**Response to reviewer comment: **The authors thank the reviewer for this important observation. In response to this comment, the manuscript has been revised to clarify both the type and manufacturer information of the polypropylene fibers and to resolve the inconsistency regarding the reported melting temperature. First, the fiber type has now been clearly specified in Section 2.1. The polypropylene fibers used in this study are monofilament polypropylene fibers with a length of 12 mm and a diameter of approximately 18 μm. These fibers were selected due to their effectiveness in improving crack resistance and reducing spalling in cementitious composites exposed to elevated temperatures. Second, the inconsistency related to the melting temperature has been corrected throughout the manuscript. The correct melting temperature of polypropylene fibers is approximately 160–170°C, which corresponds to the melting point of isotactic polypropylene reported in the literature. The previously stated value of 320–340°C in Section 3.4 was incorrect and has been removed from the revised manuscript to maintain consistency. The text in Section 3.4 has been revised accordingly to state that polypropylene fibers soften and melt at approximately 160–170°C, forming micro-channels within the cementitious matrix that facilitate vapor release and reduce internal pore pressure during heating.

 These revisions ensure consistency and improve the clarity and accuracy of the material description in the manuscript.

**5. Curing Conditions:** The manuscript states that curing occurred 'in water at 20 ± 2°C until 28 days old," but it does not clarify whether the water was lime-saturated, which is the standard method to prevent calcium leaching from mortar and influences hydration products and final strength. Additionally, it is not mentioned if the curing water was renewed regularly. These details should be included.

**Response to reviewer comment:**  In the present study, the specimens were cured in tap water maintained at 20 ± 2°C until the age of 28 days. Although lime-saturated water is sometimes recommended to minimize calcium leaching, the use of tap water for curing cementitious specimens is a common laboratory practice and has been widely adopted in many experimental studies on mortar and concrete.

 To maintain stable curing conditions, the curing water was periodically renewed, and the temperature of the curing tank was maintained within the specified range (20 ± 2°C). These details have now been clarified in Section 2.1 of the revised manuscript to improve the transparency and reproducibility of the curing procedure.

 6. **Novelty Justification and Literature Differentiation:** Although the mortar-vs-concrete distinction is presented as the novelty claim, the Introduction lacks a detailed mechanistic comparison to fully justify why this difference merits separate investigation. The literature should be cited to discuss the specific quantitative differences in paste-to-aggregate ratio, pore-size distribution, and thermal cracking mode between mortar and concrete. Additionally, in Sections 3.3 and 3.5, where results are compared with previous studies, it should be clearer whether those studies examined mortar or concrete. Each comparison ought to specify the material system used in the cited work and emphasize the mortar-specific findings of the current study.

**Response to reviewer comment: **The novelty of this study lies in providing a comprehensive experimental evaluation of polypropylene fiber–reinforced cement mortar exposed to elevated temperatures up to 600°C. While most previous studies have focused primarily on fiber-reinforced concrete, the present work specifically investigates mortar systems, which exhibit different behavior mechanisms. In addition to conventional strength measurements, this study systematically examines residual compressive strength, flexural strength, load–deflection behavior, and flexural toughness after thermal exposure. The work also identifies the best polypropylene fiber dosage for improving post-fire mechanical performance of mortar and provides detailed insights into the role of fiber melting in reducing thermal damage and enhancing residual structural integrity.

 7. **Internal Inconsistency in Compressive Strength Reporting:** In Section 3.3, it states that mortars reinforced with PP fiber 'maintain a comparatively higher strength (33–28%, respectively)' for the 0.5% and 1.5% PP mixes at 600°C. However, Table 3 shows the 1.0% PP mix retains 27.6%, and the 1.5% PP mix retains 26.5%. The 1.5% mix is not the pair being described. The text should be revised to accurately reflect the tabulated values for each mix separately and organize the performance ranking clearly.

**Response to reviewer comment: **The authors appreciate the reviewer for carefully identifying this inconsistency in the description of the residual compressive strength results. The statement in Section 3.3 has been revised to accurately reflect the values reported in Table 3.

 According to the experimental results, at 600°C, the residual compressive strength values were 33.4% for the 0.5% PP mix, 27.6% for the 1.0% PP mix, and 26.5% for the 1.5% PP mix, compared with 19.1% for the control mixture. Therefore, the 0.5% PP mixture exhibited the highest residual strength, followed by the 1.0% PP and 1.5% PP mixtures, respectively. The manuscript text has been corrected to clearly present this ranking and ensure consistency with Table 4.

 8. Dedicated Limitations and Future Work Section: The manuscript lacks a 'Limitations and Future Work' section, which is a notable omission. The following limitations should be explicitly recognized: (a) only one PP fiber geometry was tested; comparing it with basalt, steel, or hybrid systems would enhance the applicability; (b) only a single mortar mix design (w/c = 0.45, 1:3 cement to sand) was studied; (c) the highest temperature tested, 600°C, does not encompass more extreme fire scenarios reaching 800–1000°C; (d) long-term durability aspects such as carbonation, chloride resistance, and creep after thermal exposure were not evaluated; and (e) the influence of different cooling methods (furnace cooling versus water quenching) on residual properties was not addressed, although this is crucial for post-fire structural assessments.

**Response to reviewer comment:** ****The authors appreciate this valuable suggestion. Accordingly, a short “Limitations and Future Work” statement has been incorporated in the revised manuscript. The present study is limited to a single PP fiber geometry and one mortar mix design (w/c = 0.45; cement:sand = 1:3) with a maximum exposure temperature of 600°C under furnace cooling conditions. In addition, long-term durability aspects after thermal exposure were not examined. Future research will investigate alternative fiber systems (e.g., basalt, steel, or hybrid fibers), higher fire temperatures (800–1000°C), different cooling regimes, and microstructural characterization techniques (e.g., SEM and porosity analysis) to provide deeper insight into thermal damage mechanisms.

**9. Minor Typographical and Presentation Errors:** Several typographical and presentation errors need correction. The axis label 'Temretures, 0C' in Figures 5, 6, and 9 should be amended to 'Temperature (°C)'. The term 'formulation' in Section 3.5 should be changed to 'formation' ('pore formation'). The phrase 'control mia' in Section 3.4 should be corrected to 'control mix'. The label 'pp%' in Figure 4 should be standardized as 'PP fiber content (%)'. Lastly, Figure 10 (toughness) would be improved by adding a supplementary data table similar to Tables 2–5, enabling readers to easily access the exact numerical toughness values.

**Response to reviewer comment:** The authors thank the reviewer for carefully identifying these typographical and presentation issues. Also, Table 7. Flexural toughness and percentage change relative to the control mix (0% PP) at each temperature, has been added

# Reviewer report: Reviewer response for version 1

**DOI:** 10.5256/f1000research.193533.r451092

**Published:** 2026-02-05

**Authors:** öznur Biricik

**Affiliations:** 1 Bursa Uludag University, Nilüfer-Bursa, Turkey

General Evaluation

 The study investigates the effects of polypropylene (PP) fiber reinforcement (at 0%, 0.5%, 1%, and 1.5% ratios) on the fluidity and post-high-temperature strength properties of mortar mixtures. While the topic is relevant, several methodological points and technical additions are required to enhance the quality of the manuscript.

 Specific Comments

1. **Workability and Casting Methodology:** In the study, the superplasticizer dosage was kept constant across all mixtures to compare slump values. However, the significantly reduced fluidity in the 1.5% fiber-reinforced mixture directly impacts the casting quality and, consequently, the compressive strength results. It would have been methodologically sounder to **maintain a constant flow/workability** by adjusting the superplasticizer dosage for each fiber ratio before preparing the strength specimens. This would ensure that the strength loss is attributed solely to the fiber characteristics rather than poor consolidation.
2. **Reference Timeliness:** The manuscript would benefit from a more up-to-date literature review. Incorporating more citations from **2023, 2024, and 2025** will strengthen the current state-of-the-art section and demonstrate the study’s relevance to recent developments.
3. **Material Characterization:** The specific properties and technical data of the **superplasticizer** used in the mixtures should be clearly detailed in the Materials section.
4. **Microstructural Analysis (SEM):** The inclusion of **Scanning Electron Microscopy (SEM) images** is essential to support the macroscopic observations. Specifically, SEM images taken after exposure to high temperatures would provide visual evidence of the "melt-out" effect of the PP fibers and the resulting micro-channels/voids that influence the residual strength.
5. **Porosity and NDT Analysis:** To better quantify the internal damage caused by thermal stress, the authors are encouraged to perform **Ultrasonic Pulse Velocity (UPV)** tests or **Mercury Intrusion Porosity (MIP)** analysis. These methods would provide a more scientific explanation for the void formation and structural degradation observed after high-temperature exposure.

Is the work clearly and accurately presented and does it cite the current literature?

Partly

If applicable, is the statistical analysis and its interpretation appropriate?

Not applicable

Are all the source data underlying the results available to ensure full reproducibility?

Yes

Is the study design appropriate and is the work technically sound?

Yes

Are the conclusions drawn adequately supported by the results?

Yes

Are sufficient details of methods and analysis provided to allow replication by others?

Yes

Reviewer Expertise:

Concrete technology

I confirm that I have read this submission and believe that I have an appropriate level of expertise to confirm that it is of an acceptable scientific standard, however I have significant reservations, as outlined above.

# Response

**Published:** 2026-03-05

**Authors:** Sheelan Hama

**Affiliations:** Civil Engineering, University of Anbar, Ramadi, Al Anbar Governorate, Iraq

Specific Comments

1. **Workability and Casting Methodology:** In the study, the superplasticizer dosage was kept constant across all mixtures to compare slump values. However, the significantly reduced fluidity in the 1.5% fiber-reinforced mixture directly impacts the casting quality and, consequently, the compressive strength results. It would have been methodologically sounder to maintain a constant flow/workability by adjusting the superplasticizer dosage for each fiber ratio before preparing the strength specimens. This would ensure that the strength loss is attributed solely to the fiber characteristics rather than poor consolidation.

**Response to reviewer comment:** The authors agree that maintaining a constant workability by adjusting the superplasticizer dosage could help isolate the pure effect of fiber reinforcement on strength. However, in the present study, the superplasticizer dosage was intentionally kept constant for all mixtures to clearly evaluate the direct influence of polypropylene fiber content on the fresh and hardened properties of mortar. As reported in the manuscript, increasing the fiber volume fraction significantly reduced the flow diameter from 185 mm for the control mix to 125 mm for the 1.5% PP mixture, indicating a clear reduction in workability with increasing fiber content.

 “To ensure a consistent basis for comparing the influence of polypropylene fiber content on fresh and hardened properties, the dosage of the superplasticizer was kept constant for all mixtures. This approach allowed the study to directly evaluate the effect of increasing fiber volume fraction on workability and mechanical performance. It is well known that fiber addition tends to reduce the flowability of cementitious composites due to increased internal friction and higher surface area of dispersed fibers. Therefore, maintaining a constant admixture dosage enabled the observed changes in flow diameter and strength to be interpreted as a combined effect of fiber inclusion and its influence on the rheological behavior of the mortar. Nevertheless, it is acknowledged that adjusting the superplasticizer dosage to maintain constant workability could be considered in future studies to further isolate the mechanical contribution of fiber reinforcement."

 Nevertheless, the reviewer’s suggestion is appreciated, and it has been acknowledged in the revised manuscript as a recommendation for future studies, where maintaining constant workability by adjusting the superplasticizer dosage could provide additional insight into the isolated mechanical contribution of polypropylene fibers.

  

1. **Reference Timeliness:** The manuscript would benefit from a more up-to-date literature review. Incorporating more citations from 2023, 2024, and 2025 will strengthen the current state-of-the-art section and demonstrate the study’s relevance to recent developments.

**Response to reviewer comment:** The introduction and literature review sections have been revised to incorporate several recent studies published between 2023 and 2025 addressing the behavior of polypropylene fiber-reinforced cementitious materials under elevated temperatures.

1. **Material Characterization:** The specific properties and technical data of the superplasticizer used in the mixtures should be clearly detailed in the Materials section.

**Response to reviewer comment:** The Materials section has been revised to clearly describe the type and technical properties of the superplasticizer used in this study. The manuscript now specifies that a polycarboxylate-based high-range water-reducing admixture, Sika ViscoCrete-180 GS, conforming to ASTM C494 Type F, was used. Additional information regarding its specific gravity (1.07), pH range (4–6), color (light brownish), and dosage (1% by weight of cement) has been added to improve the clarity and reproducibility of the experimental methodology.

 “A polycarboxylate-based high-range water-reducing admixture (HRWR), Sika ViscoCrete-180 GS, conforming to ASTM C494 Type F, was used to improve the workability of the mortar mixtures. The superplasticizer has a specific gravity of approximately 1.07, pH value ranging from 4 to 6, and a light brownish liquid appearance. The admixture is based on modified polycarboxylate ether technology and was added at a constant dosage of 1% by weight of cement for all mixtures to maintain comparable workability conditions while isolating the effect of polypropylene fiber content on the fresh and hardened properties of the mortar.”

1. **Microstructural Analysis (SEM):** The inclusion of Scanning Electron Microscopy (SEM) images is essential to support the macroscopic observations. Specifically, SEM images taken after exposure to high temperatures would provide visual evidence of the "melt-out" effect of the PP fibers and the resulting micro-channels/voids that influence the residual strength.

**Response to reviewer comment:** The authors agree that microstructural observations using Scanning Electron Microscopy (SEM) would provide direct evidence of the microstructural changes occurring in polypropylene fiber-reinforced mortar after exposure to elevated temperatures. However, SEM analysis was not performed in the present experimental program due to laboratory limitations.

 To address the reviewer’s concern, the discussion section has been expanded to include a mechanistic explanation supported by previous SEM-based studies, which reported that polypropylene fibers melt at approximately 160–170°C, leaving behind micro-channels and voids within the cementitious matrix. These micro-channels facilitate the release of internal vapor pressure during heating, thereby reducing pore pressure buildup and mitigating explosive spalling. This mechanism contributes to the improved residual strength and toughness observed in fiber-reinforced specimens compared with the control mixture.

 In addition, it has been noted in the manuscript that future work will include detailed microstructural characterization (SEM and porosity analysis) to further investigate the internal morphology of the heated mortar and the role of fiber melting in modifying the pore structure.

1. **Porosity and NDT Analysis:** To better quantify the internal damage caused by thermal stress, the authors are encouraged to perform Ultrasonic Pulse Velocity (UPV) tests or Mercury Intrusion Porosity (MIP) analysis. These methods would provide a more scientific explanation for the void formation and structural degradation observed after high-temperature exposure.

**Response to reviewer comment:** The authors sincerely thank the reviewer for this valuable suggestion. In response to this comment, Ultrasonic Pulse Velocity (UPV) tests were conducted and incorporated into the revised manuscript to provide additional insight into the internal damage and microstructural degradation caused by elevated temperatures.

 A new subsection entitled “3.7 Ultrasonic Pulse Velocity (UPV) Analysis” has been added to the Results and Discussion section. The UPV measurements were performed on all mortar mixtures after exposure to 0, 200, 400, and 600°C, and the results are presented in Table 7.
